# ALFIN-LIKE H3K4me3 reader proteins regulate embryogenesis in *Arabidopsis*

**DOI:** 10.26508/lsa.202603835

**Published:** 2026-10-15

**Authors:** Janik Scotton, Edouard Tourdot, Marc W Schmid, Sara Simonini

**Affiliations:** 1 Institute of Plant and Microbial Biology, University of Zurich , Zurich, Switzerland; 2 MWSchmid GmbH, Glarus, Switzerland

## Abstract

Development of multicellular organisms requires the precise coordination of gene expression programs. Whereas histone modifications are key regulators of transcriptional states, how these modifications are interpreted during early development remains poorly understood. Here, we investigate the role of H3K4me3 recognition during embryogenesis in *Arabidopsis thaliana*. We identify ALFIN-LIKE (AL) proteins as essential H3K4me3 readers required for early seed development. Loss of AL function leads to severe defects in embryogenesis, including impaired asymmetric zygotic division, disrupted patterning, and loss of lineage restriction. At the molecular level, AL deficiency results in widespread transcriptional misregulation and is associated with local changes in chromatin state, including reduced H3K4me3 and a concomitant increase in H3K27me3 at a subset of loci, including key developmental and flowering-time regulators. Together, our results show that interpretation of H3K4me3 by AL proteins is required for proper gene expression during early development and reveal a context-dependent relationship between activating and repressive chromatin states, highlighting a role for H3K4me3 in shaping gene expression during plant embryogenesis.

## Abstract (precis)

The study shows that interpretation of H3K4me3 is essential for translating chromatin information into the developmental decisions that establish embryonic patterning in *Arabidopsis*.

## Introduction

Development depends on the ability of cells to establish and maintain distinct gene expression programs as they transition from pluripotent states to differentiated identities. These transitions are accompanied by dynamic changes in chromatin organization, reflecting the need to both activate lineage-specific genes and repress alternative developmental programs. Understanding how chromatin-based mechanisms contribute to the establishment of these transcriptional states remains a central challenge in developmental biology.

Histone modifications are key components of chromatin regulation and are widely associated with transcriptional activity or repression (Millán-Zambrano et al, 2022). Among these, trimethylation of lysine 4 and lysine 27 on histone H3 (H3K4me3 and H3K27me3) are among the most extensively studied marks. H3K27me3, deposited by the *Polycomb* Repressive Complex 2 (PRC2), is associated with transcriptional repression and plays essential roles in developmental regulation across eukaryotes (Cao et al, 2002; Grossniklaus & Paro, 2014). In *Arabidopsis thaliana*, multiple PRC2 complexes act at different developmental stages. The MEDEA-PRC2 (also called FIS-PRC2) complex functions specifically in the female gametophyte, early embryo and endosperm, which is a placental-like tissue that fills the seed cavity and provides nutrients to the growing embryo (Ohad et al, 1996; Chaudhury et al, 1997; Grossniklaus et al, 1998; Kiyosue et al, 1999; Köhler et al, 2003). By contrast, CURLYLEAF- and SWINGER-based PRC2 complexes govern the post-embryonic phase of the plant life, including the transition from embryo to seedling and the vernalization response (Bastow et al, 2004; Schubert et al, 2006; Bouyer et al, 2011). Genetic disruption of PRC2 results in severe developmental defects, highlighting the importance of repressive chromatin states in establishing correct gene expression programs.

In contrast, the contribution of activating histone modifications to plant development remains less well understood. H3K4me3 is deposited by Trithorax group (TrxG) proteins, several of which are encoded in the *Arabidopsis* genome. Mutations in individual TrxG components generally result in mild or context-dependent phenotypes, suggesting functional redundancy (Alvarez-Venegas et al, 2003; Pien et al, 2008). However, strong disruption of H3K4 methylation leads to severe developmental defects and embryo arrest (Aquea et al, 2010), indicating that H3K4me3 is essential for plant development. Despite this, how H3K4me3 contributes to developmental transitions, and how it functionally relates to repressive chromatin pathways, remains unclear. One approach to address this question is to investigate how H3K4me3 is interpreted. The function of histone modifications ultimately depends on proteins that recognize and bind them, linking chromatin states to transcriptional outputs. In *Arabidopsis*, several plant homeodomain (PHD) finger proteins have been shown to bind H3K4me3 (Sung et al, 2006; De Lucia et al, 2008; Molitor et al, 2014; Kim & Sung, 2017; Lai et al, 2020). Whereas their biochemical interaction with H3K4me3 is well established, their contribution to developmental processes remains incompletely understood, in part due to redundancy and potential compensatory mechanisms. As a result, how H3K4me3 recognition is translated into gene regulatory outcomes during development remains largely unresolved. This question is particularly relevant during embryogenesis, when cells undergo rapid divisions - while establishing distinct identities. During this phase, chromatin states are expected to be highly dynamic, as transcriptional programs are being established and stabilized. How activating and repressive chromatin marks are interpreted and coordinated in this context, and whether this contributes to the establishment of developmental programs, remains unknown. In this study, we investigate the role of H3K4me3 interpretation during early embryogenesis in *Arabidopsis thaliana*. We identify ALFIN-LIKE (AL) proteins, a family of plant-specific PHD domain proteins (Song et al, 2013; Molitor et al, 2014; Kayum et al, 2015; Liang et al, 2018; Peng et al, 2018; Jin et al, 2024; Su et al, 2025; Wei et al, 2015; Xu et al, 2025), as key H3K4me3 readers expressed in early embryo and seed tissues. Using higher order *al* mutants, we show that loss of AL function leads to severe defects during early seed development, including impaired asymmetric zygotic division, disrupted embryonic patterning, and altered lineage specification. At the molecular level, loss of AL function is associated with widespread transcriptional misregulation and local changes in chromatin state, including reduced H3K4me3 and increased H3K27me3 at a subset of developmental regulators. These findings indicate that interpretation of H3K4me3 is required for proper gene expression during early development and reveal a developmental context in which activating and repressive chromatin states are closely linked.

## Results

### H3K4me3 preferentially marks morphogenesis and patterning genes during seed development

**Figure 1.**
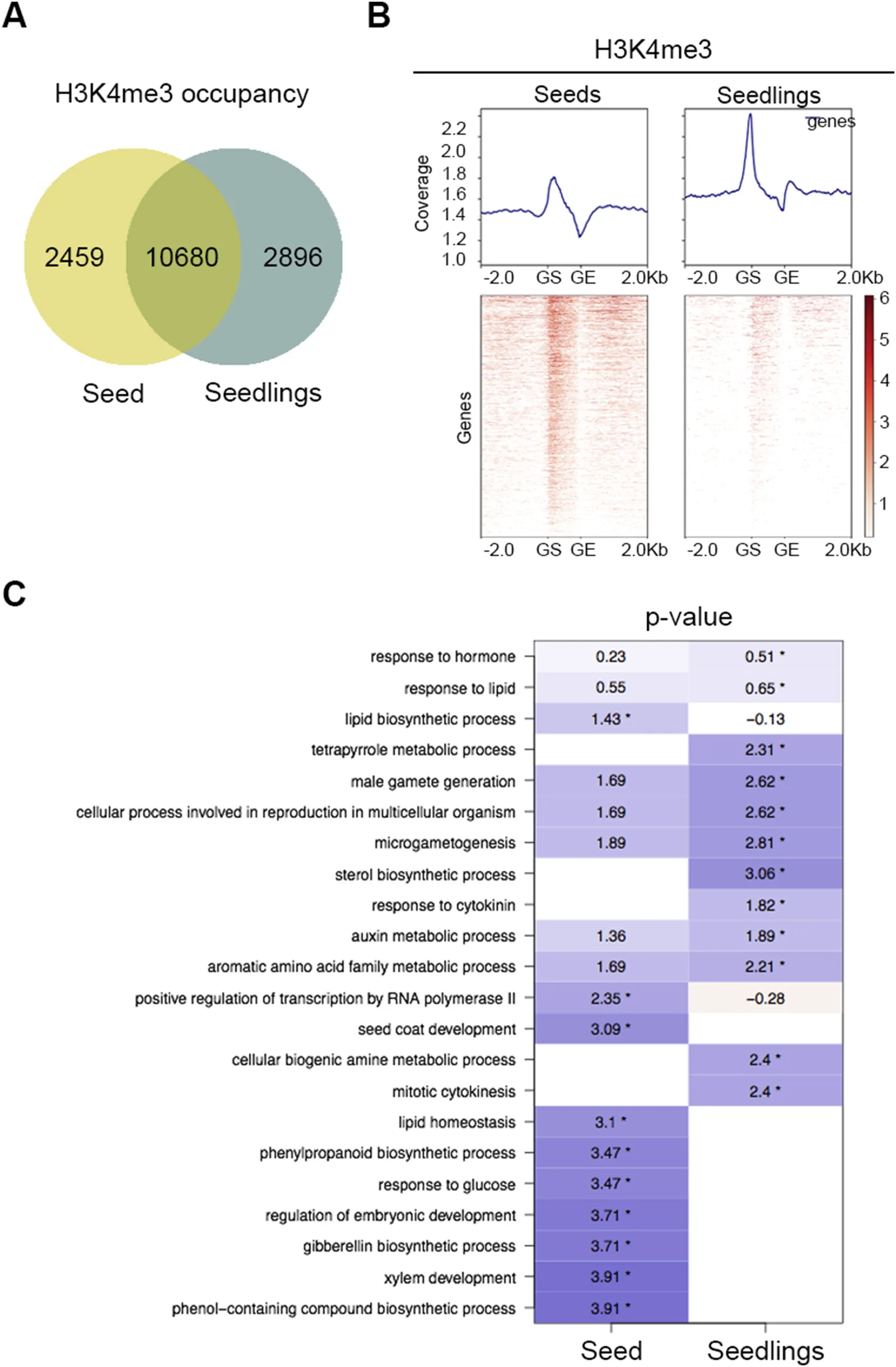
H3K4me3 occupancy differs between seeds and seedlings. **(A)** Venn diagram showing the overlap of H3K4me3 peaks identified in seeds and seedlings. **(B)** Metagene profiles (top) and heatmaps (bottom) of H3K4me3 signal across genes in seeds and seedlings. Signal is centered on gene bodies (GS: gene start; GE: gene end) and extends 2 kb upstream and downstream. **(C)** Gene Ontology (GO) term enrichment analysis of genes associated with H3K4me3 peaks in seeds and seedlings. Selected biological processes are shown, highlighting distinct functional categories enriched in each developmental stage. Values represent enrichment scores (−log10 *P*-value), with asterisks indicating significant enrichment.

H3K4me3 is broadly associated with active transcription, yet whether it plays a specific role in developmental patterning remains unclear in plants. To address this, we profiled genome-wide H3K4me3 occupancy using CUT&Tag (Fu et al, 2026) in two contrasting developmental contexts: developing seeds (4–7 d after fertilization), encompassing embryos from the globular to late heart stage, and post-embryonic seedlings, which represent a differentiated state with relatively stable transcriptional programs (Fig 1A and B and Table S1).

Table S1. CUT&Tag profiles of H3K4me3 and H3K27me3 marks in wild type, *al* quintuple and *al* sextuple mutant in seedlings and seeds.

Genome-wide comparison revealed substantial overlap in H3K4me3 occupancy at genes involved in core cellular functions, including metabolism and cytoskeletal organization (Fig 1C and Table S1). Strikingly, however, loci preferentially marked in seeds were strongly enriched for transcriptional regulators of morphogenesis and patterning, including numerous factors with established roles in embryo and endosperm development (Fig 1C and Table S1). Gene ontology analysis confirmed significant enrichment for processes such as cell fate specification, radial pattern formation, and seed development. By contrast, loci preferentially marked in seedlings were enriched for housekeeping functions as well as hormone- and environment-responsive pathways. These findings indicate that H3K4me3 is not uniformly distributed across active genes but is preferentially associated with developmental regulators during seed development, suggesting that its genomic allocation is dynamically reconfigured to support early patterning programs.

### H3K4me3 readers of the AL protein family are expressed during seed development

To investigate how H3K4me3 contributes to gene regulation during seed development, we next focused on proteins that recognize this mark, as disrupting its interpretation provides an entry point to assess its function independently of its deposition. In plants, H3K4me3 is bound by plant homeodomain (PHD) finger proteins. We therefore examined the expression of candidate H3K4me3 readers during early seed development.

**Figure 2.**
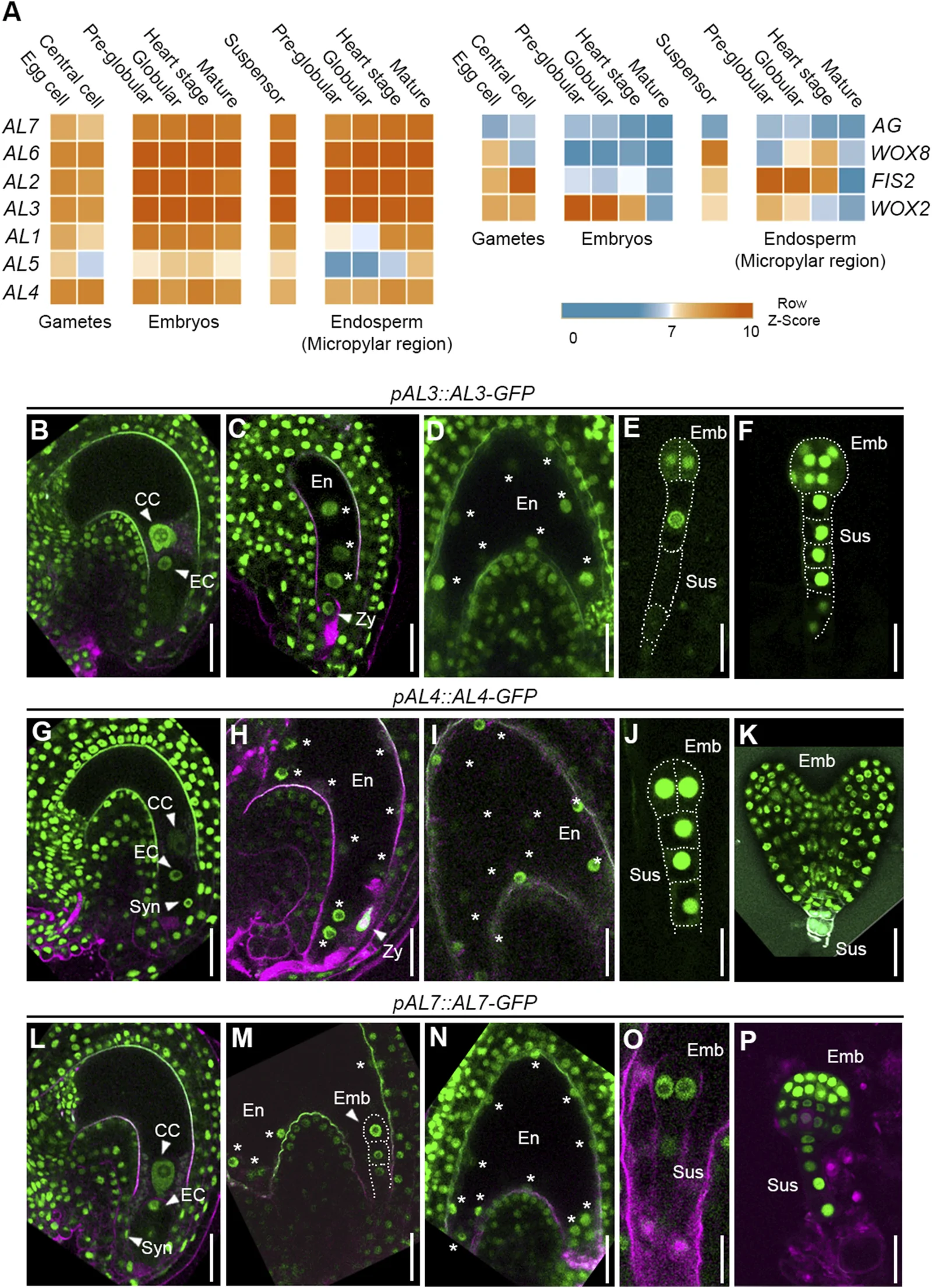
AL proteins are expressed during seed development in embryo and endosperm. **(A)** Heatmap showing the expression of *AL* genes in female gametes, during embryogenesis, and across different endosperm domains. As a comparison, expression of genes not expressed during seed development (*AG*), or specifically expressed in the embryo (*WOX8*), the endosperm (*FIS2*), or both tissues (*WOX2*), is shown. **(B, C, D, E, F)** Expression pattern of *pAL3::AL3-GFP* in the unfertilized ovule (B), zygote and early endosperm (C), proliferating endosperm (D), and early globular (E) and globular embryos (F). **(G, H, I, J, K)** Expression pattern of *pAL4::AL4-GFP* in the unfertilized ovule (G), zygote and early endosperm (H), proliferating endosperm (I), and early globular (J) and heart-stage embryos (K). **(L, M, N, O, P)** Expression pattern of *pAL7::AL7-GFP* in the unfertilized ovule (L), one-cell embryo and early endosperm (M), proliferating endosperm (N), and early globular (O) and late globular embryos (P). EC, egg cell; CC, central cell; En, endosperm; Syn, synergid cell; Sus, suspensor. Asterisks “*” label endosperm nuclei. Cell walls are stained with propidium iodide (magenta). Scale bar, b, c, f, g, h, l, k, m, p: 20 μm. d, i, n, 50 μm. e, j, o, 10 μm.

Analysis of published transcriptomic datasets revealed that all *AL* genes are expressed during seed development, in both the embryo and the endosperm across multiple stages (Fig 2A). Expression levels were comparable to those of established developmental regulators functioning in these tissues, such as *WUSCHEL HOMEOBOX 8* (*WOX8*) and *FERTILIZATION INDEPENDENT SEED2* (*FIS2*) (Fig 2A). Interestingly, *AL* transcripts were already present in the female gametes, the egg cell and the central cell, indicating that they are expressed before fertilization and may function at the earliest stages of embryogenesis.

To validate these patterns at the protein level, we generated functional *pAL::AL–GFP* reporter lines. These revealed AL protein accumulation in the egg cell and central cell, persisting after fertilization in the zygote and throughout early seed development in the embryo proper, suspensor, and endosperm (Fig 2B–P).

Together, these observations indicate that AL proteins are present across fertilization and are broadly expressed in the cell types undergoing the earliest developmental transitions during seed formation.

### AL proteins are required for embryonic patterning

**Figure S1.**
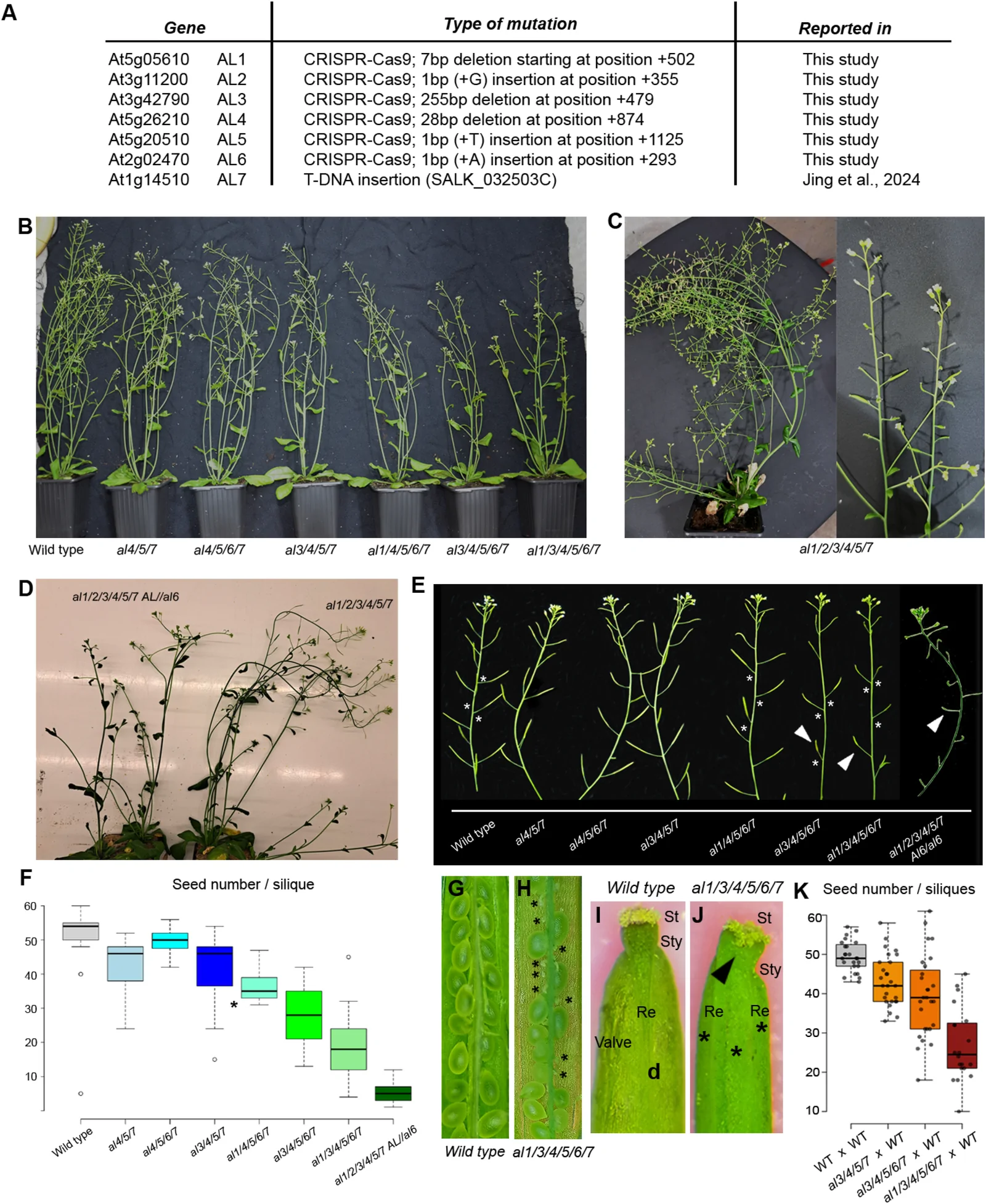
Macroscopic morphological analysis of al mutliple mutants generated using CRISPR-Cas9. **(A)** Summary of CRISPR-Cas9 generated alleles obtained for AL1, AL2, AL3, AL4, AL5 and AL6. **(B, C, D)** Overall phenotype of *al* multiple mutant plants. **(E)** Primary stems of wild type, triple, qudruple, quntuple and sextuple *al* mutants, showing reduced flower number, altered phyllotaxis (asterisks), and shorter siliques (arrow head). **(F)** Seed set of *al* multiple mutant plants, represented as the number of seeds per silique. **(G, H)** Close-up of open siliques from wild type (G) and *al* sextuple mutant (H), highlighting ovule abortions (asterisks). **(I, J)** Close-up of carpel tips from wild-type (I) and *al* sextuple mutant (J) showing an increased number of valves (asterisks) and a cleft in the style (arrow head). **(K)** Recirpocal crosses between *al* multiple mutant and wild-type pollen to assess potential female gametophytic defects, represented as number of seeds per silique.

The early expression of AL proteins in gametes and the zygote suggested a role at the onset of embryogenesis. To test whether H3K4me3 interpretation is required for early developmental patterning, we generated higher order al mutants using CRISPR–Cas9 (Fig S1A). Because the AL family comprises seven closely related members with broad and overlapping expression patterns, substantial redundancy was anticipated. We recovered multiple combinations ranging from triple to sextuple mutants, including two independent sextuple lines (*al2/3/4/5/6/7* and *al1/3/4/5/6/7*). A septuple homozygous mutant could not be obtained, and the highest-order line recovered retained one functional *AL6* allele (*al1/2/3/4/5/7 AL6/al6*), indicating that *ALs* genes collectively fulfill essential functions in plant development.

Triple and quadruple mutants were largely indistinguishable from wild type (Fig S1B). In contrast, quintuple and sextuple mutants displayed progressively severe pleiotropic defects, including delayed growth, altered flowering time, abnormal phyllotaxis, reduced fertility, and shortened siliques with frequent ovule abortion consistent with previous reports (Fig S1B–F; Su et al, 2025; Xu et al, 2025). Pistils frequently exhibited structural abnormalities, including additional valves and altered style morphology, which partially impaired fertilization (Fig S1G–J). However, manual pollination only partially restored seed set, suggesting additional defects in female gametophyte development (Fig S1K).

**Figure 3.**
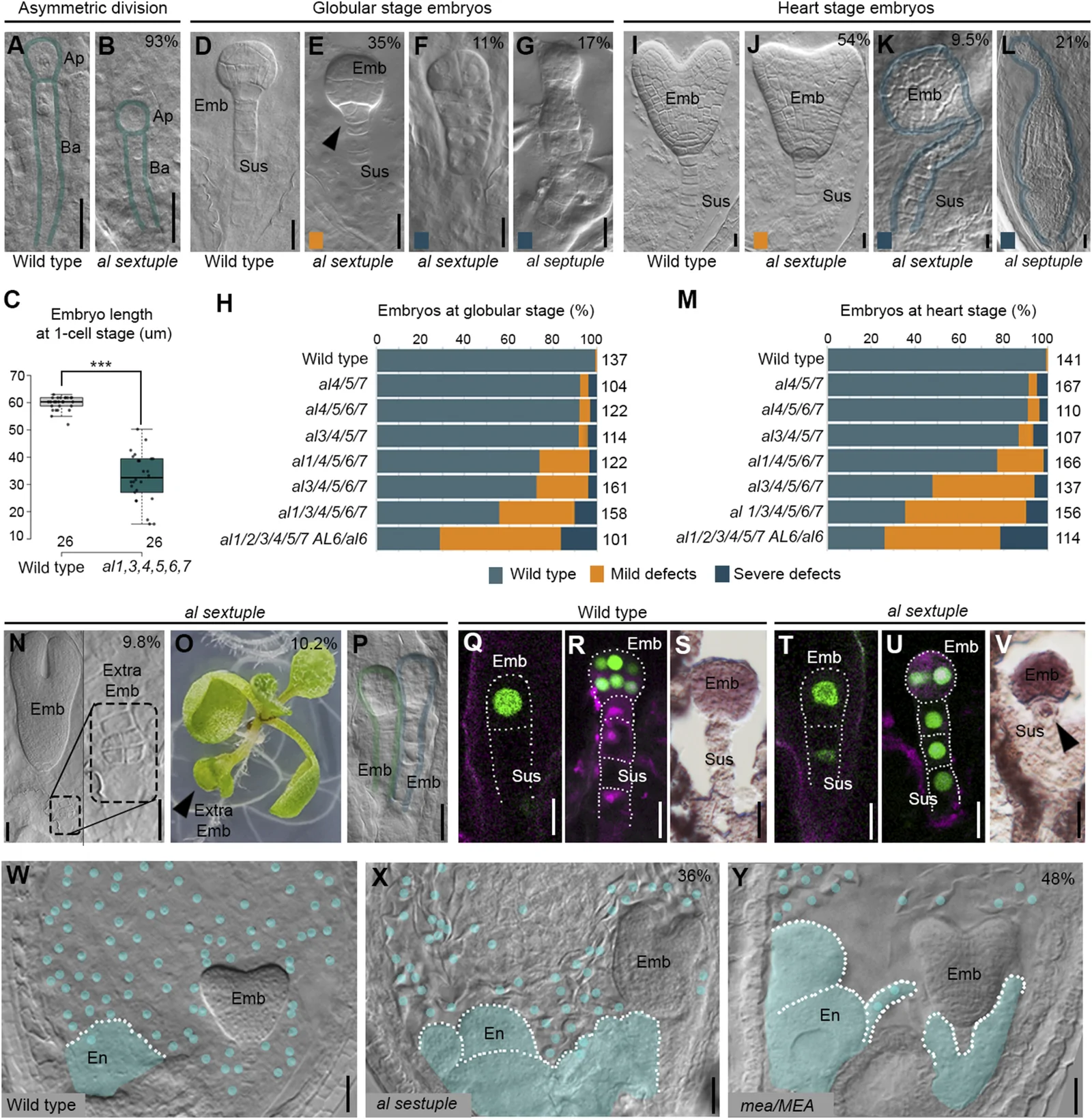
AL proteins regulate embryo and endosperm development and patterning. **(A, B)** Cleared images of wild-type (A) and *al* sextuple mutant (B) one-cell embryos. **(C)** Quantification of one-cell embryo length in wild-type and *al* sextuple mutant; ***, *P* < 0.001. **(D, E, F, G, H)** Cleared images (D, E, F, G) and quantification (H) of globular stage embryos in wild-type (D), *al* sextuple mutant (E, F; orange: mild defects; blue: severe defects), and *al* septuple mutant (G) Percentages of embryos displaying each phenotype are indicated in the top right corner. **(I, J, K, L, M)** Cleared images (I, J, K, L) and quantification (M) of heart stage embryos in wild-type (I), *al* sextuple mutant (J, K; orange: mild defects; blue: severe defects), and *al* septuple mutant (L). Percentages of embryos displaying each phenotype are indicated in the top right corner. **(N, O, P)** Additional embryos developing from *al* sextuple mutants. **(Q, R, S, T, U, V)** Expression of the embryonic marker *WOX2* in wild-type (Q, R, S) and *al* sextuple mutant embryos (T, U, V), visualized using a *pWOX2::nlsGFP* reporter (Q, R, T, U) and in situ hybridization (S, V), showing ectopic expression of *WOX2* in the suspensor of *al* sextuple embryos. **(W, X, Y)** Endosperm development in wild-type (W) and al sextuple mutant (X), compared with the *mea/MEA* mutant (Y), showing a similar phenotype. Ap, apical cell; Ba, basal cell; Emb, embryo; En, endosperm; Sus, suspensor. Cell walls are stained with propidium iodide (magenta). Scale bar,a-v 20 μm, w-y 50 μm.

**Figure S2.**
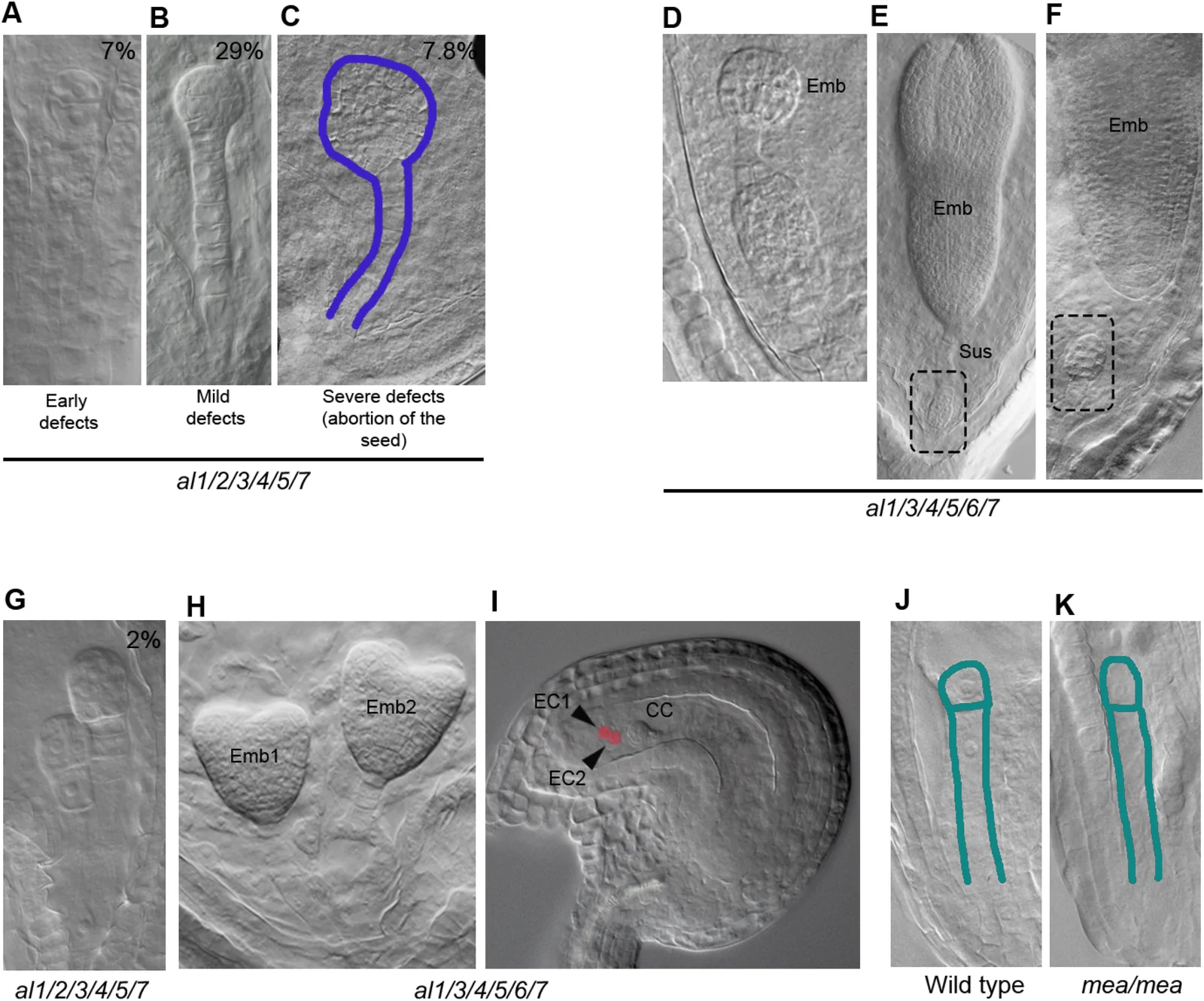
Developmental defects of *al* multiple mutants during reproductive phase and seed development. **(A, B, C)** Embryonic defects in *al* sextuple mutant. **(D, E, F)** Ectopic embryos developing from the suspensor in *al* sextuple mutants. **(G, H)** Twin embryos developing within a single embryo sac in the *al* sextuple mutant. **(I)** Clearing image of an *al*1/3/4/5/6/7 ovule exhibiting two egg cells (artificially colored in red). **(J, K)** Embryo lacking MEA activity and showing normal length.

The most striking abnormalities became evident during early embryogenesis. In *Arabidopsis*, the first zygotic division establishes apical–basal polarity and separates the embryo proper from the extraembryonic suspensor lineage. In wild type, the zygote elongates and divides asymmetrically, producing daughter cells of distinct size and fate (Fig 3A). In *al* sextuple mutants, zygotes frequently failed to elongate and instead divided more symmetrically, disrupting the earliest establishment of polarity (Fig 3A–C). Subsequent divisions were variably affected (Fig 3D–M), resulting in phenotypes ranging from distorted but viable embryos to severely disorganized cell masses lacking recognizable polarity and ultimately arresting development (Figs 3E–G and K–L, and S2A–C). Loss of polarity was accompanied by defects in lineage restriction. Suspensor cells frequently overproliferated and in some cases acquired embryonic characteristics, giving rise to ectopic embryo-like structures and occasional twin seedlings (Figs 3N–P, and S2D–I). Consistent with a breakdown in lineage identity, the embryo-specific gene *WOX2*, normally confined to the embryo proper, was ectopically activated in suspensor cells from the first zygotic division onward (Fig 3Q–V).

Endosperm development was also impaired, with reduced nuclear proliferation and abnormal nuclear morphology (Fig 3W and X). Although aspects of the *al* phenotype resemble defects reported for other chromatin regulators, including COMPASS-related and *Polycomb* factors (Fig 3Y), none have been shown to disrupt the asymmetric first zygotic division. Notably, zygotes in the *Polycomb* mutant *mea* elongated and divided asymmetrically, comparable to wild type (Fig S2J and K). This indicates that H3K4me3, but not PRC2 activity, is required for initial polarity establishment in the zygote.

Together, these findings demonstrate that AL proteins are essential for the earliest developmental transition after fertilization, linking H3K4me3 interpretation to the establishment of apical–basal polarity and proper tissue differentiation during embryogenesis, and more generally, early seed development.

### AL proteins coordinate transcriptional programs during early seed development

To investigate how AL proteins contribute to transcriptional regulation during early seed development, we performed RNA-seq on seeds collected 1–4 d after fertilization, spanning stages from the zygote to the early globular embryo, a developmental window where morphological defects becomes evident in *al* mutants. We compared wild-type seeds with *al3/4/5/6/7* quintuple and *al1/3/4/5/6/7* sextuple mutants, in which embryonic defects are progressively more severe.

**Figure 4.**
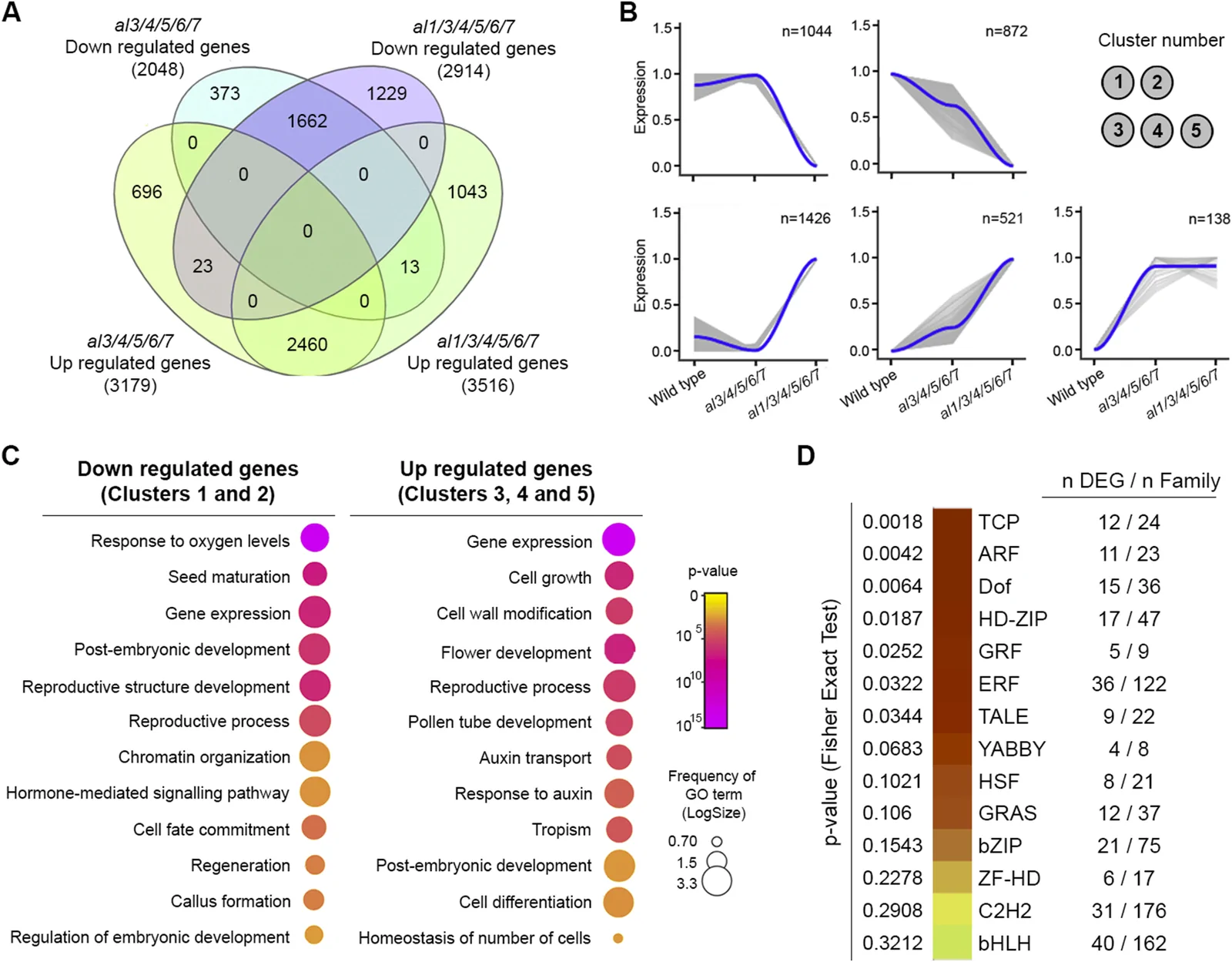
Transcriptome analysis of *al* multiple mutants reveals a close link with developmental processes and transcriptional regulation. **(A)** Venn diagram depicting the overlap in RNA-Seq analysis of wild-type, *al3/4/5/6/7*, and *al1/3/4/5/6/7* young seeds. **(B)** Cluster analysis showing five clusters with distinct expression patterns, with down-regulated genes in clusters 1 and 2 and up-regulated genes in clusters 3, 4, and 5. **(C)** GO term enrichment analysis of up-regulated genes (clusters 3, 4, and 5) and down-regulated genes (clusters 1 and 2). The color of each dot corresponds to the *P*-value, and the size of the circle represents the frequency of that particular GO term, shown as LogSize. **(D)** Enrichment of transcription factor families with deregulated expression in *al1/3/4/5/6/7* seeds. The *P*-value was calculated using Fisher’s exact test. The number of deregulated genes within each family is indicated in the top right corner.

Differentially expressed genes (DEGs) were classified as up-regulated or down-regulated (Fig 4A and Table S2) and further grouped by clustering analysis to identify distinct expression patterns (Fig 4B). Among down-regulated genes, we identified both loci showing progressive reduction from quintuple to sextuple mutants, as well as genes nearly silenced in both genetic backgrounds. Given that H3K4me3 and AL proteins are generally associated with transcriptional activation, we propose that core AL-dependent genes are predominantly represented among the down-regulated DEGs. Notably, the *Polycomb* group protein MEA, which is required for endosperm development, was strongly down-regulated in the *al* sextuple mutant, which could explain the similar endosperm defects observed in *al* and *mea* mutants (Fig 3W–Y).

Table S2. RNA-Seq analysis of seeds of *al* quintuple and *al* sextuple mutant compared with wild type.

Clustering of up-regulated genes revealed three main expression patterns: (i) genes exclusively up-regulated in the sextuple mutant, (ii) genes progressively induced from quintuple to sextuple mutants, and (iii) genes stably up-regulated in both backgrounds (Fig 4B and Table S2). Gene ontology enrichment analysis of down-regulated genes highlighted processes directly related to embryo and seed development, including cell identity acquisition and developmental patterning (Fig 4C and Table S2), consistent with the observed mutant phenotypes. In contrast, up-regulated genes were enriched for distinct biological processes, including hormonal responses – particularly auxin signaling – as well as developmental programs beyond seed formation, such as floral development and reproductive processes including pollen tube growth (Fig 4C and Table S2). We interpret these up-regulated genes as secondary transcriptional responses arising from disruption of primary AL-dependent regulatory networks.

Interestingly, the GO term “regulation of gene expression” was strongly represented among both up- and down-regulated genes, suggesting that AL proteins act at high levels within transcriptional hierarchies. Consistent with this, multiple transcription factor families were significantly enriched among DEGs, including TCP, ARF, HD-ZIP, GRF, and TALE family members – key regulators of plant morphogenesis and developmental patterning (Fig 4D and Table S2). Together, these results indicate that AL proteins are required to maintain correct transcriptional programs during early seed development, in both the embryo and the endosperm, and that their loss leads to widespread disruption of developmental gene regulatory networks.

### AL4 associates with chromatin regulatory complexes during silique development

**Figure S3.**
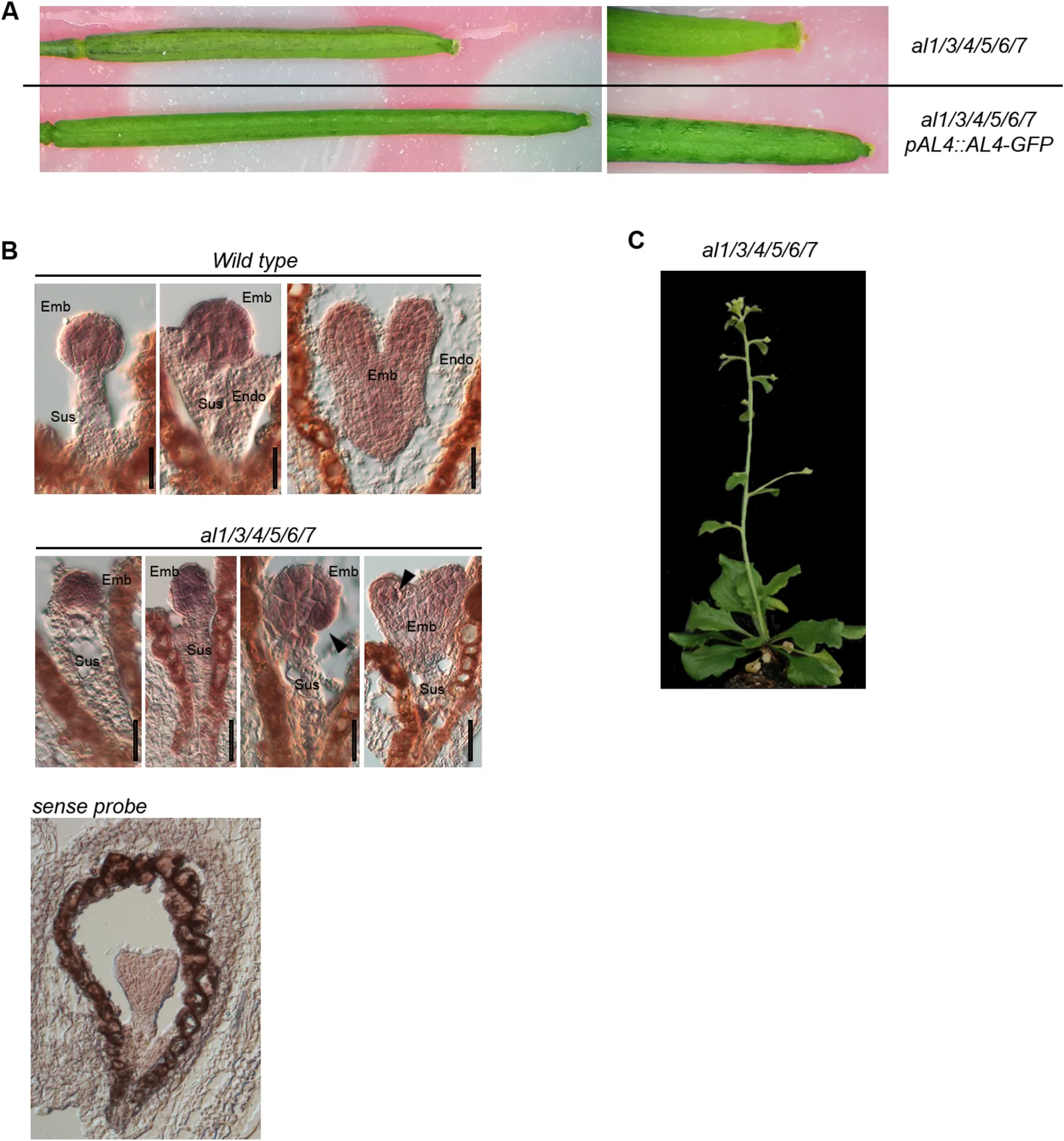
AL proteins regulate the expression of flowering regulators. **(A)** *pAL::AL4-GFP* transgene complements fruit length and pistil defects of the *al*1/3/4/5/6/7 mutant, confirming that the AL4-GFP protein is fully functional. **(B)** In situ hybridization with a *MAF* antisense and sense probe in wild type and *al* sextuple mutant embryos. **(C)** *al* sextuple mutant plant showing few enlarged rosette leaves.

Recent studies identified AL proteins as partners of chromatin remodelers and transcriptional activators (Su et al, 2025), but this was based on seedling tissue. Because of the strongest AL related phenotypes occur during seed development, we investigated whether AL proteins have tissue-specific interactomes. We therefore performed immunoprecipitation followed by mass spectrometry (IP/MS) using a functional *pAL4::AL4-GFP* complementation line expressed in developing siliques (1–4 d after fertilization). We introduced AL4-GFP into the *al1/3/4/5/6/7* sextuple mutant background, where it successfully complemented developmental defects, restoring phenotypes similar to the *al1/3/5/6/7* quintuple mutant (Fig S3A).

**Figure 5.**
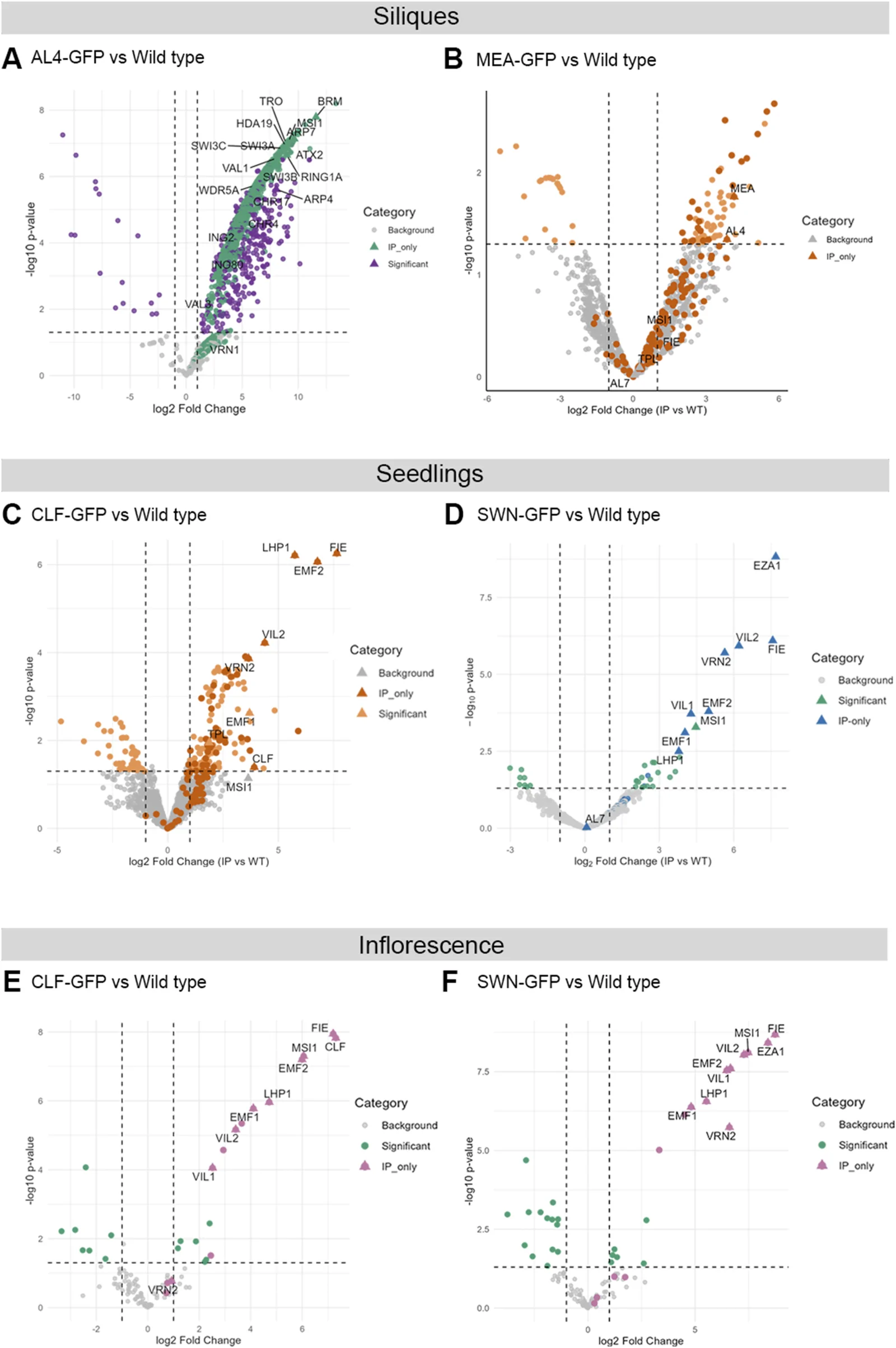
Identification of AL4 and PRC2 interactome. Volcano plots showing proteins enriched in GFP immunoprecipitation (IP) samples compared with wild-type controls across different tissues and baits. The x-axis represents log_2_ fold change (IP versus WT), and the y-axis represents −log_10_ *P*-value. Dashed lines indicate significance and fold-change thresholds. Few selected proteins annotated. **(A)** AL4-GFP versus wild type in siliques. **(B)** MEA-GFP versus wild type in siliques. **(C)** CLF-GFP versus wild type in seedlings. **(D)** SWN-GFP versus wild type in seedlings. **(E)** CLF-GFP versus wild type in inflorescences. **(F)** SWN-GFP versus wild type in inflorescences.

IP/MS analysis identified a robust AL4-associated chromatin protein network in developing siliques (Fig 5A and Table S3). As expected based on previous studies in seedlings (Su et al, 2025), AL4 co-purified with numerous factors involved in transcriptional activation and chromatin remodeling, including the Trithorax-related histone methyltransferase ATX2 and multiple SWI/SNF chromatin-remodeling components, such as BRAHMA (BRM), ARP7 and the SWI3A, SWI3B, and SWI3C subunits, consistent with a conserved role of AL proteins within activating chromatin environments. AL4 immunoprecipitates also contained proteins previously associated with transcriptional repression, including the PRC1 component RING1A (Molitor et al, 2014), the B3-domain transcriptional repressor VAL1, the histone deacetylase HDA19 and the PRC2 subunit MSI1. Together, these findings indicate that AL4 is recovered in chromatin-associated protein complexes containing both activating and repressive chromatin regulators during early silique development.

Table S3. IP/MS experiment of SWN-GFP in seedlings and inflorescences, CLF-GFP in seedlings and inflorescences, MEA-GFP in siliques and AL4-GFP in siliques. The negative control is wild type.

Reciprocal IP-MS analyses of GFP-tagged PRC2 methyltransferases likewise recovered AL proteins among MEA-associated protein complexes. In developing siliques, the seed-specific methyltransferase MEA significantly co-purified with AL4, whereas AL7 was detected but did not reach the significance threshold (Fig 5B and Table S3). By contrast, AL7 was significantly enriched in SWN immunoprecipitations (Fig 5D–F and Table S3), indicating that recovery of AL proteins is not restricted to a single PRC2-associated dataset.

Together, these results indicate that AL proteins and PRC2 complexes are functionally connected and coexist within shared chromatin-associated environments. This is consistent with a model in which activating and repressive chromatin regulators might operate in close proximity during embryogenesis.

### AL proteins constrain chromatin state dynamics during early seed development

**Figure 6.**
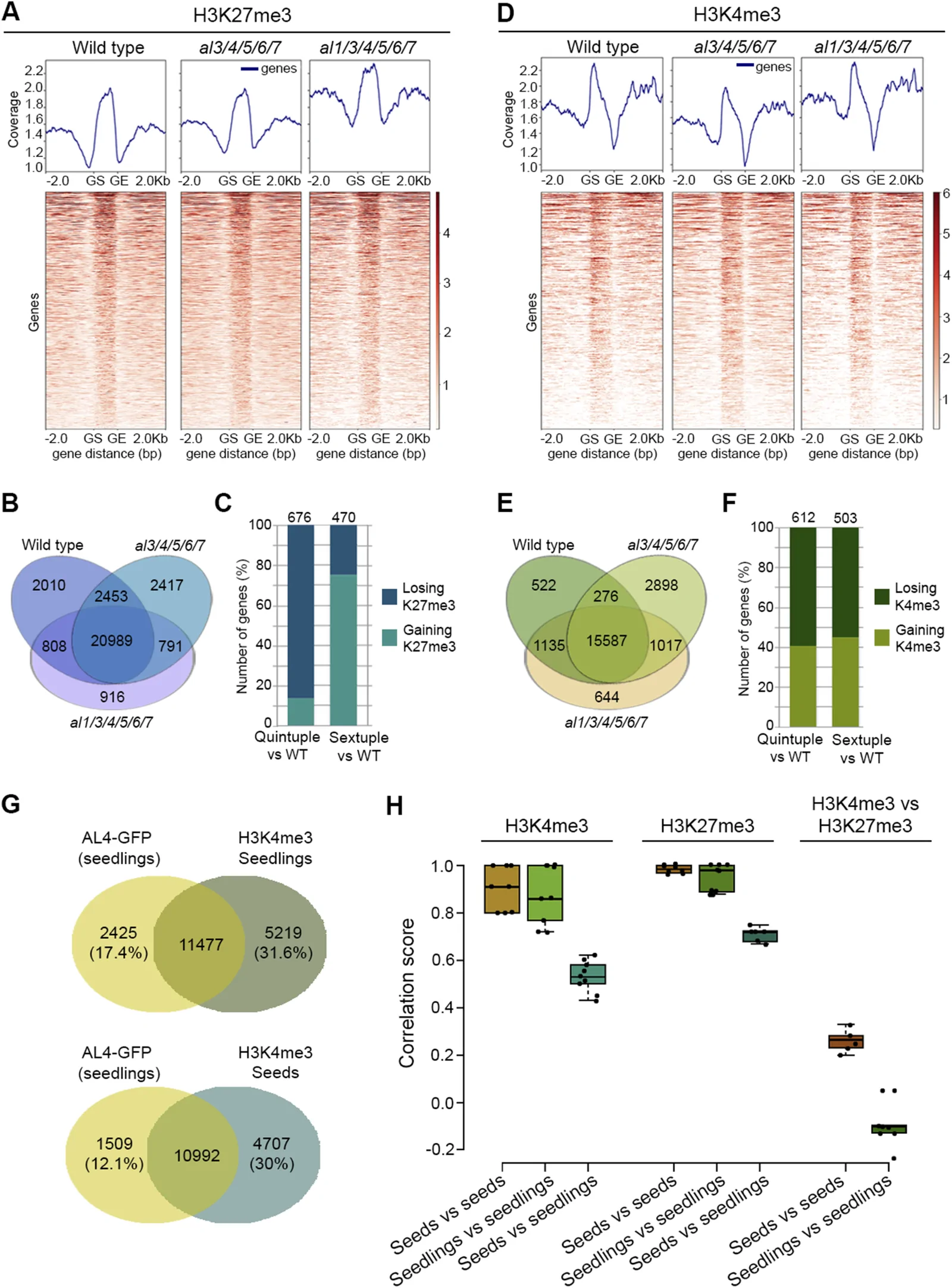
AL proteins maintain H3K4me3 patterns without globally affecting H3K27me3 distribution. **(A)** Heatmaps and metagene profiles showing H3K27me3 occupancy across gene bodies and flanking regions in wild-type, *al3/4/5/6/7*, and *al1/3/4/5/6/7* young seeds. GS: gene start; GE: gene end. **(B)** Venn diagram showing overlap of genes marked by H3K27me3 in wild-type, *al3/4/5/6/7*, and *al1/3/4/5/6/7* young seeds. **(C)** Proportion of genes gaining or losing H3K27me3 in *al3/4/5/6/7* and *al1/3/4/5/6/7* mutants relative to wild type. **(D)** Heatmaps and metagene profiles showing H3K4me3 occupancy across gene bodies and flanking regions in wild-type, *al3/4/5/6/7*, and *al1/3/4/5/6/7* young seeds. **(E)** Venn diagram showing overlap of genes marked by H3K4me3 in wild-type, *al3/4/5/6/7*, and *al1/3/4/5/6/7* young seeds. **(F)** Proportion of genes gaining or losing H3K4me3 in *al3/4/5/6/7* and *al1/3/4/5/6/7* mutants relative to wild type. **(G)** Overlap between AL4–GFP ChIP–seq peaks identified in seedlings (Su et al, 2025) and H3K4me3-marked genes defined by CUT&Tag in seedlings (top) and developing seeds (bottom). **(H)** Correlation analysis of CUT&Tag datasets showing relationships within and between H3K4me3 and H3K27me3 profiles in seedlings and seeds. H3K4me3 and H3K27me3 profiles are highly correlated within each tissue, whereas correlations between the two marks are negative in seedlings but shift toward positive values in seeds.

Pull-down experiments with AL4 identified associations with PRC2 components, raising the possibility that AL proteins might recruit PRC2 to chromatin. To test this hypothesis, we profiled H3K27me3 distribution in wild-type, *al3/4/5/6/7* quintuple, and *al1/3/4/5/6/7* sextuple mutants mutant seeds using CUT&Tag (Fig 6A and B, and Table S1). Contrary to a recruitment model, global H3K27me3 levels and genomic distribution were not reduced in *al* mutants and were, if anything, modestly increased compared with wild type (Fig 6C and Table S1). Similar observations have been reported in seedlings (Su et al, 2025), collectively arguing against a role for AL proteins as canonical PRC2 recruiters.

Given that AL proteins are established readers of H3K4me3, we next examined whether their loss affects H3K4me3 landscapes during early seed development. We therefore performed CUT&Tag profiling of H3K4me3 in developing seeds from wild type, *al3/4/5/6/7* quintuple, and *al1/3/4/5/6/7* sextuple mutants (Fig 6D–E, and Table S1).

Genome-wide analysis revealed that loss of AL function results in localized alterations in H3K4me3 levels. Both mutant backgrounds displayed loci with reduced H3K4me3 as well as loci with gains in H3K4me3 signal (Fig 6F and Table S1).

Importantly, H3K4me3 enrichment remained strongly centered at transcription start sites (TSS) in both wild-type and mutant backgrounds, with no evidence for redistribution along gene bodies. This indicates that the overall genomic distribution of H3K4me3 is preserved in *al* mutants. Together, these results show that AL proteins do not broadly alter global H3K4me3 distribution, but are associated with locus-specific changes in H3K4me3 levels.

Consistent with this role, AL4–GFP ChIP–seq peaks identified in seedlings (Su et al, 2025) overlapped extensively with H3K4me3-enriched regions defined by CUT&Tag in both seedlings and developing seeds (Fig 6G and Table S1). Despite the distinct developmental contexts, the majority of AL4-bound loci corresponded to H3K4me3-marked regions, supporting the conclusion that AL proteins occupy a conserved subset of H3K4me3-enriched chromatin domains across developmental stages.

Comparison of H3K4me3 and H3K27me3 distributions across developmental stages revealed distinct relationships between the two marks. In seedlings, H3K4me3 and H3K27me3 showed predominantly negative correlations, consistent with their largely mutually exclusive genomic distribution. In contrast, in seeds, the two marks exhibited positive correlations across genomic regions, indicating a higher degree of co-occurrence (Fig 6H and Table S1). Because these analyses were performed on whole seeds, which comprise multiple cell types with distinct chromatin states, this increased overlap cannot distinguish between co-occurrence of the two marks within individual cells and their presence in different cell populations. Nevertheless, it indicates that the genomic distributions of H3K4me3-and H3K27me3-associated regions are considerably less segregated in seeds than in seedlings, consistent with the dynamic chromatin landscapes of early seed development.

### AL proteins limit accumulation of H3K27me3 at flowering time regulators

**Figure 7.**
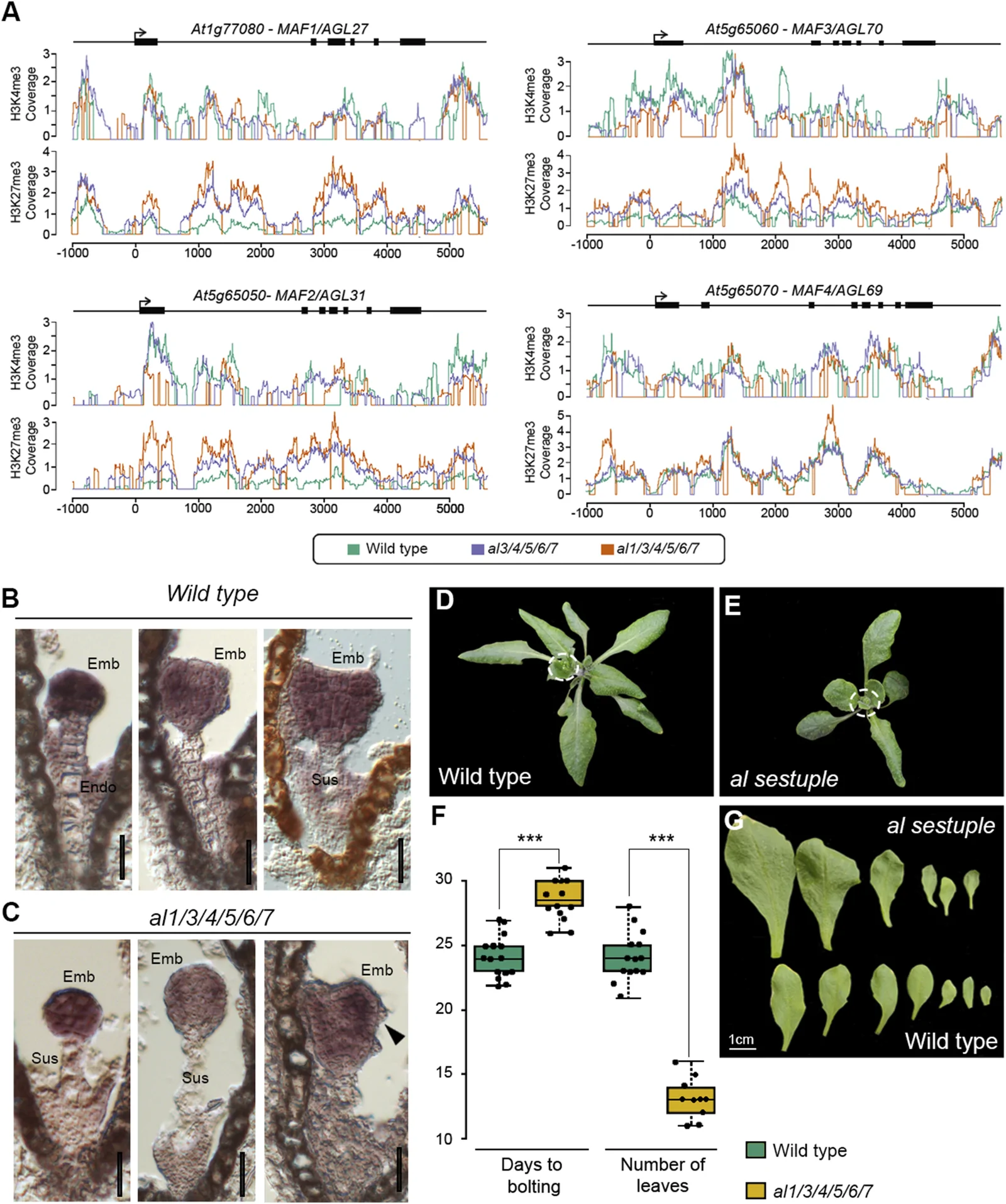
AL function links H3K4me3 and H3K27me3 at flowering regulators and impacts development. **(A)** Genome browser views of H3K4me3 and H3K27me3 profiles at *MAF1–4* loci in wild type, *al3/4/5/6/7*, and *al1/3/4/5/6/7* developing seeds. Reduced H3K4me3 is accompanied by increased H3K27me3 in *al* mutants. **(B, C)** In situ hybridization with a *MAF* antisense probe in embryos from wild type (B) and *al1/3/4/5/6/7* (C). Mutants show altered expression (arrowhead). **(D, E)** Representative wild-type (D) and *al1/3/4/5/6/7* (E) adult plants at the same developmentl age. Al sextule mutant produces less leaves before bolting than wild type, but bolting is slightly delayed. **(F)** Flowering time measured as days to bolting and number of rosette leaves at bolting for wild-type and *al1/3/4/5/6/7*. ***, *P* < 0.001. **(G)** Rosette leaf size comparison between wild-type and *al1/3/4/5/6/7* mutant. Emb, embryo; Sus, suspensor; Endo, endosperm. Scale bars, 50 μm.

Our data indicate that loss of AL function is associated with changes in chromatin state at a subset of loci, including flowering-time regulators. In *al* multiple mutants, the *MAF* genes, a clade of MADS-box transcription factors that repress flowering (Ratcliffe et al, 2003), were down-regulated in both quintuple and sextuple backgrounds (Table 2), accompanied by a modest reduction in H3K4me3 and a pronounced increase in H3K27me3, particularly in the sextuple mutant (Fig 7A and Table S1). These changes were concentrated within the first intron, a known regulatory region controlling flowering time (Fu et al, 2005; Costa & Dean, 2019).

Flowering repressors such as *MAF* genes are reactivated during embryogenesis to ensure proper developmental timing in the next generation (Sheldon et al, 2008; Tao et al, 2019). To assess whether this expression is affected in *al* mutants, we performed in situ hybridization using a probe detecting multiple *MAF* transcripts. In wild-type embryos, *MAF* expression was uniformly strong from the globular stage onwards (Figs 7B and S3B). In *al1/3/4/5/6/7* embryos, expression remained initially comparable, even in morphologically affected embryos, but appeared more heterogeneous and patchy at later stages (Figs 7C and S3B), indicating increased variability in expression patterns relative to wild type.

A similar relationship between reduced H3K4me3 and increased H3K27me3 at flowering regulators has been reported in other mutants affecting H3K4me3 deposition. For example, loss of H3K4me3 at the *FLC* locus in *atx* mutants is associated with increased H3K27me3 (Pien et al, 2008; Tamada et al, 2009; Yun et al, 2012), and comparable effects have been described at *MAF* genes too (Berr et al, 2015). These observations suggest that the coupling between reduced H3K4me3 and increased H3K27me3 at these loci is not unique to AL function, but may represent a broader feature of chromatin regulation at developmental genes, particularly of those involved in flowering. Moreover, similar chromatin alterations at flowering-time regulators have been reported in seedlings of *al* mutants. Analysis of published datasets (Su et al, 2025) shows that *MAF* loci are associated with AL proteins and display reduced H3K4me3 together with increased H3K27me3 in post-embryonic tissues (Su et al, 2025). The observation of comparable chromatin changes at these loci in both embryos and seedlings indicates that the effects of AL loss are not restricted to early developmental stages, but are also present at later stages of development.

Given the reported association between AL proteins and flowering time (Su et al, 2025), we next quantitatively assessed flowering behavior in the *al1/3/4/5/6/7* sextuple mutant using both days to bolting and rosette leaf number at the time of floral transition. Consistent with the previous report (Su et al, 2025), sextuple mutants bolted slightly later than wild type. However, mutant plants also produced substantially fewer but enlarged rosette leaves before flowering (Fig 7D–G). This indicates that mutant plants reach the floral transition after producing fewer leaves, despite taking longer to do so, consistent with a slower rate of leaf initiation (increased plastochron) rather than a simple shift in flowering time.

Together, these observations indicate that loss of AL function affects developmental timing and is associated with altered chromatin states at flowering-related genes across developmental stages.

## Discussion

The regulation of gene expression by histone modifications has been extensively studied, yet the functional contribution of individual marks to development remains incompletely understood, particularly in plants. Here, we investigated the role of H3K4me3 during embryogenesis through the analysis of AL proteins, a family of plant-specific H3K4me3 readers.

In animals, histone modifications such as H3K27me3 are deposited in the oocyte and shape the early embryonic transcriptome (Zenk et al, 2017), whereas chromatin remains highly plastic in the zygote. During early embryogenesis, H3K4me3 is established before H3K27me3, suggesting that activating chromatin states can influence subsequent repressive marking (Akkers et al, 2009; Liu et al, 2019). In plants, however, the extent to which histone modifications are inherited from the gametes and contribute to early embryogenesis remains largely unknown as the chromatin landscapes of the *Arabidopsis* egg cell and zygote have not yet been resolved. Genetic evidence indicates that activating and repressive chromatin marks contribute differently to early development. Whereas embryos lacking PRC2 function initially develop normally (Ohad et al, 1996; Grossniklaus et al, 1998; Bouyer et al, 2011; Simonini et al, 2021), disruption of H3K4me3 deposition leads to early embryonic arrest (Aquea et al, 2010), indicating a more immediate requirement for H3K4me3. Consistent with this, loss of AL function results in defects at the earliest stages of embryogenesis, including impaired asymmetric zygotic division and disrupted apical–basal patterning. These defects are followed by abnormal tissue organization and altered cell identities, highlighting the importance of H3K4me3 interpretation during the establishment of developmental programs.

One prominent phenotype of *al* mutants is the formation of embryo-like structures from the suspensor, reflecting a failure to restrict embryogenic potential to the embryo proper (Liu et al, 2015; Meinke, 2020). Auxin signaling plays a central role in this process (Rademacher et al, 2012), as well as the correct expression of embryonic trasncription factors (Radoeva et al, 2020). Consistent with this, genes related to auxin transport and signaling are enriched in the *al* mutant transcriptome. Together, these findings indicate that AL proteins are required to establish and maintain proper cell fate specification during embryogenesis.

At the chromatin level, loss of AL function is associated with locus-specific changes in H3K4me3 and H3K27me3 at developmental regulators, including flowering-time genes. Similar relationships between reduced H3K4me3 and increased H3K27me3 have been reported in mutants affecting H3K4me3 deposition (Pien et al, 2008; Tamada et al, 2009; Yun et al, 2012; Berr et al, 2015), as well as in seedlings of *al* multiple mutants (Su et al, 2025). Rather than reflecting a global redistribution of chromatin states, our data indicate that this interplay occurs in a locus-specific manner and is observed across developmental stages.

Although AL proteins predominantly associate with factors linked to H3K4me3 deposition and transcriptional activation, we also detected interactions with PRC1 components in our IP/MS datasets, consistent with previous reports identifying PRC1 subunits as AL interactors (Molitor et al, 2014) and with recent work linking AL proteins to PRC1-associated pathways (Su et al, 2025; Xu et al, 2025). These observations support a functional connection between AL proteins and PRC1-mediated chromatin regulation. In contrast, interactions with PRC2 components were only weakly detected in our analysis, arguing against a direct recruitment mechanism and instead suggesting that activating and repressive regulators act in close proximity at shared loci.

Notably, AL proteins have also been reported to interact with the H3K27me3 demethylase RELATIVE OF EARLY FLOWERING 6 (REF6) (Su et al, 2025), which was also identified in our IP/MS datasets. This raises the possibility that the increased H3K27me3 observed in *al* mutants may, at least in part, reflect reduced H3K27me3 removal. In this context, AL proteins could contribute to maintaining lower H3K27me3 levels at specific loci by facilitating REF6-mediated demethylation.

These observations raise the possibility that the chromatin changes observed in *al* mutants may involve indirect mechanisms, such as altered H3K27me3 removal or modulation of PRC1-dependent chromatin states, rather than direct effects on PRC2 activity.

Together, our results show that interpretation of H3K4me3 by AL proteins is required for proper developmental patterning during embryogenesis. More broadly, our findings highlight a context in which activating and repressive chromatin states are closely linked at key regulatory loci during early development. Future work will be required to determine how these interactions are established and maintained, and how they contribute to developmental trajectories from embryogenesis to later stages of plant growth.

## Materials and Methods

### Plant material and growth conditions

Seeds were sown on half-strength MS media (1/2 MS salt base [Carolina Biologicals], 1% sucrose, 0.05% MES, 0.8% Phytoagar [Duchefa], pH > 5.7 with KOH), stratified for 3–4 d at 4°C in the dark, then moved to long-day conditions (8 h dark at 18°C, 16 h light at 22°C, 70% humidity). At the four-leaf stage, seedlings were transplanted to soil and grown under the same conditions. Marker lines and mutants used: *pCLF::CLF-GFP* in *clf-29*, *pSWN::SWN-GFP* in *swn-4* (Shu et al, 2019), *pMEA::MEA-GFP* in *mea-1* (Simonini et al, 2021). AL7 mutant allele (SALK_032503C) was obtained from NASC (Jing et al, 2025). Reciprocal crosses were performed by emasculating stage 11 flowers, allowing 24 h for ovule maturation, and manually pollination with the desired pollen.

### CRISPR-Cas9 strategy to create multiple *al* mutants

Guide RNA sequences for *AL* knockouts were designed using CHOPCHOP v3, selecting three guides per gene. Primers were ordered from IDT and used in PCR with pAGM9037 as the template. Two constructs to mutate *AL4* as been assembled following the cloning strategy in Wang et al (2015) with single gRNA (AL4-CR862 and AL4-CR902) and used to transform the homozygous mutant for the *AL7* gene. T1 plants have been selected on Hygromicin. To create a higher level of mutants, the gRNA chosen are: 44,45 targeting *AL1*; 46,48 targeting *AL2*; 49,51 targeting *AL3*; 55,57 targeting *AL5*; 59, 60 targeting *AL6*. The gRNAs selected have been assembled following the cloning strategy as in Grüzner et al (2021) in the final plasmid pAGM65879. T1 plants have been selected for BASTA resistance. A second construct as been created targeting only *AL2* using the gRNA 47 and 48 following the strategy for two gRNA plus the selection gene redseed as in Grüzner et al (2021). T1 plants have been screen for red fluorescence in the seed coat. Each construct was then introduced in *Arabidopsis* by *Agrobacterium* GV3101-mediated transformation. Primer sequences are in listed in Table S4.

Table S4. Primers used in this study.

### Vectors

For construction of the ALs GFP-tagged lines (*pAL3::AL3-GFP*, *pAL4::AL4-GFP* and *pAL7:.AL7-GFP*), their respective genomic sequence were amplified from Col-0 genomic DNA using the couple of primers listed in the Table S4.

For *pAL3::AL3-GFP*, the genomic region of the gene *AL3* has been amplified with the primers: olAL3-1 (−4413 from ATG) and AL3-2; olAL3-3b and olAL3-4b to amplify the gene region; olAL3-5b and olAL3-6b to amplify the region 875 bp downstream the gene. Fragments were subcloned in pJET1.2 (Thermo Fisher Scientific) and assembled together with the GFP plasmid pSB279 in the backbone pSB280 with the enzyme SapI following Golden Gate cloning strategy.

For *pAL4::AL4-GFP*, the genomic region of the gene AL4 has been amplified with the primers: olAL4-1 (−2979 from ATG) and AL4-2; olAL4-3 and olAL4-4 to amplify the gene region; olAL4-5 and olAL4-6 to amplify the region 294 downstream the gene. Fragments were subcloned in pJET1.2 (Thermo Fisher Scientific) and assembled together with the GFP plasmid pICSL50008 in the backbone pSTB140 with the enzyme BsaI following Golden Gate cloning strategy.

For the *pAL7::AL7-GFP* construct, the genomic region of the *AL7* gene was amplified using primers SAS239, SAS240, SAS241, and SAS242. An endogenous BsaI restriction site was removed by amplifying the genomic region in two fragments and introducing a point mutation via the primers. The resulting fragments were subcloned into pJET1.2 (Thermo Fisher Scientific) and subsequently assembled together with the GFP-containing plasmid pICSL50008 into the backbone pSTB140 using BsaI-mediated Golden Gate cloning.

The *pWOX2:3xGFPnls* includes 3.5 Kb of the *WOX2* promoter as reported in Ueda et al (2011), driving a 3xGFP-NLS marker. The promoter has been amplified in two parts using the primers pWOX2-_1 pWOX2_2 and pWOX2-_3 pWOX2_4, to remove an internal BsaI site. The resulting fragments were subcloned into pJET1.2 (Thermo Fisher Scientific) and subsequently assembled together with the GFP-containing plasmid pICSL50008 into the backbone pSTB140 using BsaI-mediated Golden Gate cloning.

The destination vector pSTB140 is a modified version of the Golden Gate low copy binary vector pAGM53451 (Grützner et al, 2021). For our purpose, we inserted in the pAGM53451 vector the RedSeed selector marker at position three, whereas at position two we inserted the lacZ gene adapted to be an acceptor for Level 1 Golden Gate modules. For AL4, the final constructs has been introduced by floral-dip (Clough & Bent, 1998) in the *al1/3/4/5/6/7* sextuple mutant. Plants that showed recovered phenotypes like the *al1/3/5/6/7* quintuple mutant were selected and brought to homozygosity. Primer sequences are in listed in Table S4.

### Clearing

Siliques were fixed overnight in ethanol:acetic acid (9:1), then transferred to 70% ethanol, seeds/ovules were dissected and mounted on a drop of chloral hydrate solution and left to clear for a few hours or overnight, and imaged using a Zeiss DMR microscope with DIC filters and a Leica Flexacam C3 LSR camera, with objective 20× and 40×.

### Imaging of GFP-tagged lines

ALs-GFP marker lines were imaged with a inverted Leica TCS SP8 Multiphoton microscope in photon counting mode set at 10% power, using the MP1 shutter set at 988 nm. RLD detectors were used to acquire spectra related to GFP and PI emissions. Objective used for both ovules and embryos is a 63× water immersion objective, Zoom 1.25×, Pinhole 1AU. Ovules and seeds were mounted in 7% glucose with 10 μg/ml Propidium Iodide.

### In situ hybridization

The digoxygenin-labeled antisense RNA probes for *WOX2* and *MAFs* were generated by in vitro transcription according to the instructions provided with the DIG RNA labeling kit (SP6/T7; Roche) using a plasmid containing the individual probes sequence as template (for sequences, see Table 4). Developing siliques were collected at the desired time points and fixed in FAA fixative (formaldehyde, acetic acid, ethanol) o/n, then moved in ethanol 70% and into an ASP200 embedding machine for embedding (Leica Microsystems GmbH). The samples were then sliced in 8-μm sections with a RM2145 Leica microtome (Leica Microsystems GmbH) and then hybridized as described previously, with strong formaldehyde washes, as described in Dreni et al (2007). Images were taken with a Zeiss DMR microscope equipped with differential interference contrast (DIC) filters and Leica Flexacam C3 LSR camera.

### Protein immunoprecipitation coupled with liquid chromatography tandem mass spectrometry (LC-MS/MS)

For immunoprecipitation followed by liquid chromatography-tandem mass spectrometry (IP LC-MS/MS) experiments, *pCLF::CLF-GFP* and *pSWN::SWN-GFP* seedlings at 14 d post-germination were harvested and stored at −80°C until further processing. In addition, for IP LC-MS/MS experiments involving *pCLF::CLF-GFP*, *pSWN::SWN-GFP* whole inflorescence were collected. For *pMEA::MEA-GFP* and for *pAL4::AL4-GFP* lines, siliques at 1–4 d after fertilization were collected. All samples were flash-frozen in liquid nitrogen and stored at −80°C until further use.

For the immunoprecipitation step, we followed the protocol reported in Wendrich et al (2017). The frozen tissue was ground in liquid nitrogen for 30 min, and the resulting powder was collected in a 50 ml Falcon tube and stored at −80°C. The following day, the frozen powder was resuspended in EB + buffer (50 mM Tris–HCl, pH 7.5, 150 mM NaCl, 1% NP-40, protease inhibitor) at a ratio of ∼30 ml EB+ buffer per 10 g of powder. The homogenate was filtered through Miracloth (Merck KGaA) and sonicated using a Bioruptor (five cycles of 15 s on and 15 s off, medium power). Samples were incubated on ice for 30 min before centrifugation at 39,000*g* for 15 min at 4°C to remove debris. The supernatant was further filtered using a 40 μm cell strainer. For immunoprecipitation, anti-GFP beads were added at a ratio of 1:500 and incubated with rotation at 4°C for 2 h. GFP-Trap Magnetic Agarose (ChromoTek) were used for CLF-GFP, SWN-GFP, and AL4-GFP immunoprecipitations. Anti-GFP MicroBeads (Miltenyi Biotec) were used for MEA-GFP samples. Immunoprecipitations were performed according to the manufacturerʼs protocol. Beads were washed with EB+ buffer and eluted in ABC buffer (50 mM NH_4_HCO_3_). Eluted samples were further processed and prepared for LC-MS/MS analysis at the Functional Genomics Center Zurich starting with an On-beads digestion with Trypsin, and precipitation of the peptides with 0.1% trifluoroacetic acid (TFA) in 50% acetonitrile. All fractions were combined and dried before further processing. Peptide concentrations were estimated using the Lunatic UV/Vis absorbance spectrometer (Unchained Lab). Digested peptide samples were dissolved in 3% acetonitrile with 0.1% formic acid and analyzed using a liquid chromatography-tandem mass spectrometry (LC-MS/MS) workflow. Peptides were separated on an M-class Ultra-Performance Liquid Chromatography (UPLC) system and analyzed using an Orbitrap mass spectrometer (Thermo Fisher Scientific). Peptide concentrations were re-evaluated using the Lunatic UV/Vis absorbance spectrometer before LC-MS injection. Basic quality control of raw LC-MS data, including total ion chromatogram (TIC), base peak chromatogram (BPC), lock-mass correction, and internal retention time (iRT) peptide signal assessment, was performed using the rawDiag-shiny application (Trachsel et al, 2018).

Label-free quantification (LFQ) with match-between-runs (MBR) was performed using the FragPipe-RESOURCE workflow executed within the b-fabric app 295. Data processing was conducted using the Philosopher command-line interface (CLI [Da Veiga Leprevost et al, 2020]), and LFQ was carried out with the IonQuant software tool (Yu et al, 2021). Label-free quantification (LFQ) intensity data were processed in R using a custom pipeline. Protein intensities and spectral counts were extracted and merged across replicates. Protein entries mapping to the same gene were collapsed to a single entry by selecting the protein with the highest mean LFQ intensity across all replicates as a representative.

LFQ intensities were log2-transformed, and missing values were imputed per sample using a minimal value approach (minimum observed value − 1). No global normalization was applied. For datasets generated across multiple experimental batches, batch effects were corrected using the limma remove BatchEffect function.

Differential enrichment between IP samples and wild-type controls was assessed using linear modeling with the limma package, followed by empirical Bayes moderation. Proteins were classified as significantly enriched based on a *P*-value <0.05 and a log2 fold change >1.

In parallel, spectral count data were used to define “IP-only” proteins, requiring detection in at least two IP replicates and absence in all control samples. Results were visualized using volcano plots displaying log2 fold change versus −log10 *P*-value, with IP-only and selected candidate proteins highlighted.

### RNA-seq

The RNA-Seq has been performed on isolated seeds from 1 to 4 d after fertilization. Siliques were fixed on a double-sided tape, open along the replum with a fine needle, seeds gently scraped off with a needle and immediately placed in an Eppendorf filled with RNAlater buffer (Thermo Fisher Scientific) and placed on ice. After maximum 1 h of collection, seeds were precipitated by centrifugating the tubes at 500*g* for 2 min at RT, the supernatant removed, and the seed pellet stored at −80°C until the day of extraction. Total RNA was extracted using the Vazyme FastPure Universal Plant Total RNA Isolation Kit, following the manufacturerʼs instructions. RNA integrity and concentration were assessed using the TapeStation 4200 and RNA ScreenTape Analysis. DNA contamination was removed by treatment with DNase I (Invitrogen TURBO DNase). First-strand cDNA synthesis was performed using Invitrogen SuperScript II Reverse Transcriptase with oligo(dT) primers and 5 μg of total RNA. Second-strand synthesis followed the Gubler & Hoffman method (Gubler & Hoffman, 1983). Strand-specific libraries were prepared using the Nextera XT DNA Kit. Sequencing was carried out at the Functional Genomics Center Zurich (FGCZ) on an Illumina NovaSeq X Plus 1 Lane 10B Flowcell. cDNA and concentration were assessed using the TapeStation 4200 and DNA and High Sensitivity ScreenTape Analysis. All bioinformatics analyses were conducted on a local machine running Ubuntu 24.04.1 LTS (Noble Numbat) within a Conda 24.9.2 environment. Quality control was performed with FastQC v0.12.1 and MultiQC v1.25.2, whereas adapter trimming was conducted using Fastp v0.24.0 (Chen et al, 2018). Read mapping and gene expression quantification were performed with STAR v2.7.11b (Dobin et al, 2013). The *Arabidopsis thaliana* reference genome used was Araport11 (Cheng et al, 2017). Differential expression analysis was conducted in R v4.4.3 using DESeq2 v1.46.0, applying a log2FC >0.5 and an adjusted *P*-value <0.01 as significance thresholds. Gene ontology (GO) term enrichment analysis was performed using PLAZA 5.0 (Van Bel et al, 2022). DE genes were clustered by hierarchical clustering using z-scores with an Euclidean distance and Wardsʼs agglomeration method.

### CUT&Tag

For the CUT&Tag, we followed the protocol described in Fu et al (2026). Antibodies used in this study are: Rabbit anti-H3K27me3 (#9733; Cell signaling technology, dilution ratio 1:50); Rabbit anti H3K4me3 (c15410003; Diagenode, dilution ratio 1:150); Mouse anti-H3 (39064; Active Motif, dilution ratio 1:150). The generated libraries were pooled and sequenced on an Illumina NovaSeq X Plus sequencer. For bioinformatic data processing, short reads generated in this study were deposited at NCBI Sequence Read Archive (SRA, www.ncbi.nlm.nih.gov/sra) and are accessible through the accession numbers PRJNA1066606 and PRJNA1248163. Following removal of sequencing adapters and low-quality bases with fastp (version 0.23.4 with the options -l 30 -a CTG​TCT​CTT​ATA​CAC​ATC​T) paired-end reads were aligned to the *Arabidopsis thaliana* reference genome (TAIR10) using bowtie2 (version 2.4.5 with the options --local --very-sensitive --no-mixed --no-unal --no-discordant --phred33 -I 10 -X 700, [Langmead & Salzberg, 2012]). Only reads with a minimal alignment quality of 10 were kept. Duplicate reads were removed with Picard tools (version 3.1.1, broadinstitute.github.io/picard). Peak calling was done with MACS2 using the matching H3 libraries as background control (version 2.2.7.1 with the options -f BAMPE -g 119146348 --nomodel -q 0.05 --broad --broad-cutoff 0.1, [Zhang et al, 2008]). Peaks different replicates were merged with multovl (version 1.3 with the option -m 1 -u, [Aszódi, 2012]) and reads per peak were counted with featureCounts (version 2.0.6 with the options -O -p, [Liao et al, 2014]). Peaks were intersected with the *Arabidopsis thaliana* gene annotation (Araport11) using 3/1.5 kb up-/down-stream flanking sequences and the program mapGenomicRangesOverlap (github.com/MWSchmid/mapGenomicRanges).

For differential binding analysis, variation in counts per peak was analyzed with a general linear model in R with the package DESeq (version 1.38.3; [Love et al, 2014]) according to a factorial design with all two explanatory factors combined into a single factor (Schmid, 2017). Specific conditions were compared with linear contrasts. Within each comparison, *P*-values were adjusted for multiple testing (Benjamini-Hochberg), and peaks with an adjusted *P*-value (false discovery rate, FDR) below 0.05 and a minimal log2 fold-change (i.e., the difference between the log2 transformed, normalized sequence counts) of 1.5 were considered to be differentially bound. Normalized sequence counts were calculated accordingly with DESeq2 and log2(x+1) transformed.

For functional characterization (Gene Ontology), we tested for enrichment of gene ontology (GO) terms using topGO (version 2.50, [Alexa et al, 2006]) in conjunction with the GO annotation available through biomaRt (Durinck et al, 2009). Analysis was based on gene counts using the “weight” algorithm with Fisher’s exact test (both implemented in topGO). A term was identified as significant if the *P*-value was below 0.01. For data visualizations (to visualize coverage along a genomic stretch), coverage was depth-normalized with bedtools genomecov (version 2.31.0, [Quinlan & Hall, 2010]) and BedGraphs were converted to BigWigs with bedGraphToBigWig (version 2.10, [Kent et al, 2010]). Coverage heatmaps were drawn using deeptools as described in the tutorial (version 3.5.1, [Ramírez et al, 2014]). If more than 1,000 were available for display, sites were downsampled randomly to 1,000 sites.

## Supplementary Material

Reviewer comments

## Data Availability

The raw RNA-Seq and CUT&Tag dataset generated in this work can be accessed at NCBI-SRA under the numbers PRJNA1066606 and PRJNA1248163. The mass spectrometry proteomics data have been deposited to the ProteomeXchange Consortium via the PRIDE partner repository with the dataset identifier PXD081074. All plant material and lines generated in this work are fully available upon request.
