# Supplementary material for: ALFIN-LIKE H3K4me3 reader proteins regulate embryogenesis in Arabidopsis
Source: Life Sci Alliance. 2026 Oct 15;10(1):e202603835. doi: 10.26508/lsa.202603835 (PMC13673680; doi:10.26508/lsa.202603835)
Supplement: Supplementary file 1 [file LSA-2026-03835_review_history.pdf]

ALFIN-LIKE H3K4me3 Reader Proteins Regulate Embryogenesis in Arabidopsis

Janik Scotton, Edouard Tourdot, Marc Schmid, and Sara Simonini
DOI: https://doi.org/10.26508/lsa.202603835

Corresponding author(s): Sara Simonini, University of Zurich

Review Timeline:

Submission Date:	2026-07-05
Editorial Decision:	2026-07-10
Revision Received:	2026-09-25
Accepted:	2026-09-29

Scientific Editor: Tim Fessenden

Transaction Report:

(Note: With the exception of the correction of typographical or spelling errors that could be a source of ambiguity, letters and reports are not edited. The original formatting of letters and referee reports may not be reflected in this compilation.)

Please note that the manuscript was previously reviewed at another journal and the reports were taken into account in the decision-making process at *Life Science Alliance*.

Reviews

Review #1

Report for Author:
Unfortunately, I do not find that the manuscript has substantially improved. In addition to my continuing concerns regarding the interaction data, as detailed below in comments 1 and 2, many of the conclusions remain overstated. Also the novelty of the study is limited in light of two recent publications that have addressed both the mechanism of AL function and the functional requirement of AL proteins during vegetative and reproductive development, including flowering-time control (Su et al., 2025; Xu et al., 2025). Although these studies are cited, the manuscript does not clearly explain what new insight the present work provides relative to this existing knowledge.
1. Related to Comment 1: Rather than generating higher-quality data to address the concern, the authors have reanalyzed the existing dataset. However, even after this reanalysis, the proposed interaction between AL4 and PRC2 subunits is not convincingly supported. The claimed interactions do not appear to pass the significance threshold. Moreover, the interaction data for AL4 could not be properly assessed because the relevant proteins were not annotated in the Supplementary table. Although the authors state that the AL-PRC2 interaction is not the main focus of the study, they nevertheless present and discuss these data in the manuscript. Given the quality and limitations of the dataset, I do not think this is justified.
2. Related to Comment 2: My concern remains the same as for comment 1. The data provided do not justify discussion of this interaction. In addition, the statement in the response letter "Instead, we interpret the IP/MS results as reflecting the presence of these factors in overlapping chromatin-associated environments, particularly during early seed development" is unclear. It is not evident what specific conclusion the authors intend to draw from this interpretation, nor how it is supported by the data.
3. Related to Comment 3: The additional reporter lines are a useful addition. However, a negative control is required to distinguish the weak signals observed in the egg and central cell from background signal.
4. Page 3: The conclusion that "H3K4me3 is not uniformly distributed across active genes, but is preferentially associated with developmental regulators during embryogenesis" is not supported by the data presented. To substantiate this conclusion, the authors would need to test whether the occurrence of H3K4me3 is independent of, or not simply correlated with, transcriptional activity.
5. The transcriptome analysis was performed on seeds at 1-4 DAP, and the authors state that this stage is "before the most pronounced morphological defects arise in al mutants." However, based on Figure 3h, embryo arrest occurs at the globular stage, which corresponds to the 1-4 DAP window.
6. Page 11: The authors used siliques for the IP-MS experiment, but then conclude that "IP/MS analysis revealed a robust AL4-associated chromatin interactome in developing seeds." Because the input material was whole siliques, conclusions specifically about seeds cannot be drawn.
7. Page 13: Any inferences about the co-occurrence of H3K4me3 and H3K27me3 should be removed or substantially toned down. The authors used silique tissue, which contains multiple distinct cell types. Therefore, the data do not allow reliable conclusions about co-occurrence of these marks in a biologically meaningful way.
8. Page 15: The claimed more heterogeneous and patchy expression of MAF genes at later stages is not apparent from the data provided.
9. Finally, the late-flowering phenotype of al mutants has already been reported previously by Su et al. (2025). This should be clearly acknowledged, and the manuscript should avoid presenting this observation as a novel finding.

Review #2

Report for Author:
The manuscript has improved significantly. The authors have addressed all my concerns and most of the issues raised by other reviewers. In my opinion, the manuscript is suitable for publication in this journal.

Review #3

Report for Author:
In this new version of the manuscript the authors removed the exaggerated claims they made in the original version (tug or war, stochastic variation on gene expression, interaction with PRC2) that were critiqued by myself and the other reviewers. The authors chose to remove them rather than providing additional experimentation to support their claims. The revised manuscript is an interesting, yet somewhat descriptive characterization of the function of AL proteins during embryogenesis; crucially, it does not add to our understanding of what AL proteins are doing mechanistically and molecularly. The experiments are now technically sound and the remaining conclusions justified. However, the manuscript still lacks focus. In summary, due to the lack of mechanistic insights, the manuscript would be better suited for a more specialized journals.

Review #1

Report for Author:
This manuscript investigates a potential interaction between the H3K4me3 readers ALFIN-LIKE (ALs) and the FIS-Polycomb Repressive Complex 2 (PRC2) in Arabidopsis thaliana. The authors performed immunoprecipitation followed by mass spectrometry (IP-MS) using the PRC2 subunit MEDEA, and among numerous unvalidated interaction partners, they chose to focus on AL proteins. They generated multiple al gene mutant combinations, which exhibit abnormal embryo development. Furthermore, they profiled H3K4me3 and H3K27me3 in wild-type and al mutant seeds, proposing that reduced H3K4me3 in the mutants leads to a redistribution of H3K27me3. However, the manuscript lacks sufficient evidence to support these conclusions. The data quality is insufficient, essential experimental controls are missing, and the analyses are not robust enough to substantiate the claims.
Further Comments:
1. The statistical treatment of the IP-MS data is inadequate. It remains unclear how AL proteins were prioritized as candidates. ALs were identified in only one of four replicates, which severely questions the reliability of this interaction.
2. The proposed interaction between ALs and MEDEA requires independent validation, such as co-immunoprecipitation.
3. Line 161: Figure 1b does not convincingly demonstrate expression of AL genes in the endosperm.
4. Figure 2J: The criteria used to define "mild" and "strong" developmental defects are not provided.
5. Figure 2I: Conclusions about WOX2 expression should be validated using a WOX2 reporter line.
6. Figure 5B, D: This figure lacks clarity. The term "occupancy" is undefined, and the number of biological replicates and statistical analyses are not described. The al pentuple mutant appears more affected than the hexuple, which is counterintuitive and questions the reproducibility of the data.
7. Figure 5E: Sample labels are missing, making the figure uninterpretable.
8. The proposed antagonistic relationship between H3K4me3 and H3K27me3 is not experimentally tested. Are the same loci affected?
9. How do the authors link the proposed physical interaction of the FIS-PRC2 with the observed phenotypes of al mutants?

Review #2

Report for Author:
The manuscript provides evidence for an epigenetic "crosstalk" between activating and repressive chromatin marks, highlighting the AL protein family's role as H3K4me3 readers that limit

H3K27me3 spreading. This work has the potential to address a knowledge gap in plant epigenetics. The authors have integrated IP/MS, RNA-Seq, CUT&Tag, and in situ hybridisation data, which offers a multidimensional perspective. They have also generated higher-order mutants, which enable the functional validation of AL function. The major weakness is that the study provides limited mechanistic insight, and this should be reflected in the conclusion reached. For example, although AL proteins bind H3K4me3 and correlate with gene activation in developing embryos, the biochemical or structural basis of this interaction is underexplored. Similarly, it is unclear how ALs distinguish specific target sites or whether they actively recruit the different co-factors in embryos. The "tug-of-war" model between H3K4me3 and H3K27me3 is interesting but lacks temporal chromatin profiling or co-occupancy ChIP experiments to validate competition at the same loci. Similarly, the definition "stochastic epigenetic state" (Line 477) needs more experimental justification as cell-to-cell heterogeneity isn't directly quantified. Some methodological limitations (e.g., lack of cell-type specificity in CUT&Tag from whole seeds) are not discussed.

Nevertheless, the work is interesting in its own right and could significantly improve with some modifications:

+ Typographic errors:
Line 62: "trasnition" → "transition"
Line 183: "Septuple al mutant have been shown" → "Septuple al mutants have been shown"
Line 198: "indipendent" → "independent"
Line 269: "a further lost" → "a further loss"
Line 371: "Altoghether" → "Altogether"
Line 487: "unidirectiona" → "unidirectional"
Figure 2 legend: "developmenrt", "exprssion", "indicartes" → should be "development", "expression", "indicates"
There is an inconsistent gene and protein description (e.g., ALs, al, AL proteins)- perhaps standardise to improve clarity

+ Inconsistencies:
Line 177: "ALs perform essential function" replace by "perform an essential function"
Line 378: "suggesting a potential role in maintaining epigenetic memory" clarify if this is a new function or an extension.

+ Figures:
Figure legends are sometimes incorrectly embedded in the text.
Figure 2(l-m): Refers to subpanels L and M, but uses lower-case 'l', which can be confused with '1'.
Figure 5a could be better shown as an enrichment heatmap (compared to WT), considering transcriptional states (for different expression quantiles).
Figure 5f could have included IGV views of representative states for differentially expressed genes
Figure 6f is referenced in the legend but not clearly in the figure.

+ References:
Line 143: "(Tian et al., 2003; Wu et al., 2000))" - Double closing parentheses.
Some citations use full names, others abbreviate inconsistently (e.g., "Grossniklaus and Paro, 2014" vs. "Bouyer et al., 2011").

Review #3

Report for Author:
In this manuscript Scotton et al report a function for the ALFIN H3K4m3-reader proteins in plant development, more precisely embryogenesis and flowering time. They also suggest they function in the dynamic interplay between H3K4m3 and the polycomb repressive complex 2 (PRC2)- mediated H3K27me3. This manuscript complements our understanding of this reader protein family and provides novel aspects. The manuscript is well written and combines developmental analysis with epigenome profiling in seeds. While the experimental data provided is sound, some of the drawn conclusions are not sufficiently backed by experimental evidence as detailed below. The

manuscript has a tendency to report many different (isolated) findings (embryonic phenotypes, flowering phenotype, MAF regulation) and interesting ideas ("tug-of-war"), without tying things into a coherent picture. In addition, the findings of a recent (and cited) publication (Su et al 2025) may suggest alternative explanations in some parts and this needs to be incorporated into the current model.

Specific comments:

1) The idea that ALFINs protect H3K4me3 regions from invading H3K27me3 is very interesting. So far, this is shown on a "count" basis (genes gaining or losing modifications). It would be helpful to follow this up showing average gene plots of the different groups. It also needs to be clarified whether the modification "spreads" from neighboring regions that are enriched in H3K27me3 (as the heading in line 444 suggests) or whether it is newly laid down in the genes that loose H3K4me3. The MAF screenshots in Fig. 6 are only of limited help. To me the gain in H3K27me3 is limited and very localized, and the loss in H3K4me3 appears even smaller.

2) It is a vey exciting idea that H3K4me3/ALFIN mediates robust gene expression levels and that stochastic changes appear in the mutant. The in situ hybridization pictures are suggestive but not convincing. It looks like the expression in the heart stage embryo (6d) may simply be stronger in the inner cell layers of the cotyledons. Similarly, this pattern can be seen in (6c late heart stage embryo). The authors should provide more (convincing) pictures. Also, a sense probe as negative control is missing. It would be nice to link this phenotype also to genes relevant for embryo development, such as the mentioned WOX2 and others. This would strengthen the conclusion of enhanced stochastic variation in gene expression in alfin mutants.

3) While FLC expression is reset during early embryogenesis, there is no evidence that embryonic FLC expression is crucial for flowering time control. The discussion leaves a wrong impression and needs correction. It is thus a long stretch from a possibly reduced (?) MAF expression in early embryos to a late flowering phenotype. The authors need to show the altered expression level in seedlings. Su et reported epigenomic analysis of relevant modifications in seedlings of alfin mutants. Did the authors check whether they hypothesis for MAF gene expression is supported by those data sets?

It is an interesting question whether developmental time is counted in days or in leaves. To conclude that reduced embryonic MAF expression and early flowering fits together is cherry picking, as with the same right you could say the plants are late flowering. In this respect, an increased plastochron is interesting, given that the rosettes over all do not look smaller - judging from Fig EV1b. Additional phenotypic information would be useful. The legend of this Fig should also have a size bar and specify the age and growing conditions of the plants.

The suggested misexpression of MAFs and the flowering phenotype however remain at the stage of correlation, as any number of flowering regulators might similarly or even more clearly misexpressed. - I understand that it would require to stack a maf quadruple mutant onto the alfin sextuple mutant to test this, so I am not suggesting it.

4) Fig 5a-c: H3K4me3 is typically enriched at the TSS. The squeezing of gene bodies in Fig 5a and c in not suited to judge whether there is mislocalization of the modification in alfin mutants.

5) The manuscript would be strengthened considerable if it was known which of the genes that alter chromatin modifications are direct targets of ALFINs. While it may be difficult to globally determine ALFIN-bound genes, CHIP-seq for the 1-2 most relevant ALFINs should be done to be able to clarify whether the affected genes are direct target genes.

6) tug-of-war is a catchy phrase. I am not convinced it is appropriate here, as the effect of loss of H4K27me3 on K4me3 is not studied.

7) A role in robustness (reducing stochastic variation) in gene expression has been reported for the Paf1c complex (Fal et al, 2019; doi:10.1242/dev.173377) . Maybe ALFINs are involved with Paf1c?

8) Su et a 2025 report a connection between ALFIN and PRC1/H2A ub, as well as a direct interaction with the K27me3 demethylase REF6. The latter is also in the list of ALFIN interactors presented here. Could this be a major mechanism of antagonizing H3K27me3? Could the effects on PRC2/H3K27me3 be indirectly mediated or supported by its effect on PRC1? None of this is discussed in this manuscript although it is highly relevant for the interpretation of the data.

Small comments:

size bars are missing in multiple photographs.

Fig 4d: what do the colors mean? Please add legend.

Fig5e: Specify what values are displayed and heatmap colors.
l. 426: bivalent chromatin combines both modifications, so they are not always mutually exclusive. Please discuss.
l. 423: a shift/redistribution is only seen in the sextuple mutant, please rephrase
Fig3: typo in title
l. 395: loss - lost
l. 488/89: References within word.

Response to Reviewers' comments

We thank the editor and the reviewers for their careful evaluation of our manuscript and for their constructive comments. We have revised the manuscript accordingly and provide a detailed, point-by-point response below. Our responses are indicated in blue.

Referee #1:

This manuscript investigates a potential interaction between the H3K4me3 readers ALFIN-LIKE (ALs) and the FIS-Polycomb Repressive Complex 2 (PRC2) in Arabidopsis thaliana. The authors performed immunoprecipitation followed by mass spectrometry (IP-MS) using the PRC2 subunit MEDEA, and among numerous unvalidated interaction partners, they chose to focus on AL proteins. They generated multiple al gene mutant combinations, which exhibit abnormal embryo development. Furthermore, they profiled H3K4me3 and H3K27me3 in wild-type and al mutant seeds, proposing that reduced H3K4me3 in the mutants leads to a redistribution of H3K27me3. However, the manuscript lacks sufficient evidence to support these conclusions. The data quality is insufficient, essential experimental controls are missing, and the analyses are not robust enough to substantiate the claims.

Further Comments:
1. The statistical treatment of the IP-MS data is inadequate. It remains unclear how AL proteins were prioritized as candidates. ALs were identified in only one of four replicates, which severely questions the reliability of this interaction
We have now re-analyzed the IP/MS data and provide statistical enrichment values in the revised manuscript. We also recognize that the structure and wording of the previous version may have given the impression that we were claiming a strong and biologically central direct interaction between PRC2 and AL proteins. This was not our intention. In the revised manuscript, we no longer present the study as being centered on a direct PRC2–AL interaction. Instead, we have restructured the Results and Discussion to make clear that our main conclusion is that AL proteins and PRC2-associated factors can be found in overlapping chromatin-associated environments during seed development. Where interactions were detected, they were weak and not consistently recovered across datasets, and we therefore do not interpret them as evidence for a stable or biologically dominant interaction under our growth conditions and in the developmental stages analyzed. Accordingly, we have revised the text throughout the manuscript to clarify this point and to align our interpretation with the strength of the data.

2. The proposed interaction between ALs and MEDEA requires independent validation, such as co-immunoprecipitation.
We acknowledge that the previous version of the manuscript may have given the impression that we were proposing a direct and functionally relevant interaction between PRC2 and AL proteins. This was not our intention. In the revised manuscript, we have clarified that we do not claim a stable or direct interaction between AL proteins and PRC2. Instead, we interpret the IP/MS results

as reflecting the presence of these factors in overlapping chromatin-associated environments, particularly during early seed development. Importantly, our functional data show that loss of AL function does not lead to a global defect in H3K27me3 deposition, consistent with previous findings (Su et al., 2025). This argues against a model in which AL proteins directly recruit or regulate PRC2 activity. Given this, and the lack of strong or reproducible interaction signals in our datasets, we do not consider additional protein–protein interaction assays essential to support our conclusions. We have revised the manuscript accordingly to better align our claims with the strength of the data.

3. Line 161: Figure 1b does not convincingly demonstrate expression of AL genes in the endosperm.
We have reanalyzed AL3, AL4, and AL7 reporter lines across seed development and now provide additional images that more clearly demonstrate expression in endosperm nuclei. We note that at early stages, the endosperm is syncytial and nuclei are distributed throughout the seed cavity rather than being tightly packed. As a result, nuclei are often observed at the periphery of the cavity, close to the integuments, which may have made their identification less clear in the previous version. The revised figure includes additional images and improved annotation to better highlight endosperm expression.

4. Figure 2J: The criteria used to define "mild" and "strong" developmental defects are not provided.
We have clarified the criteria used for phenotype classification both in the main text and in the figure itself. In addition, we provide representative images for each category, which are color-coded consistently with the quantification charts. This allows a direct visual association between the defined categories and the observed phenotypes.

5. Figure 2I: Conclusions about WOX2 expression should be validated using a WOX2 reporter line.
In the revised manuscript, we provide improved *in situ* hybridization data which more clearly show ectopic *WOX2* expression in the suspensor of *al* sextuple embryos. In addition, we generated a *pWOX2::NLS-3xGFP* reporter line and introduced it into both wild-type and *al* sextuple backgrounds. The reporter analysis is consistent with our *in situ* data: in the *al* sextuple mutant, *pWOX2::NLS-3xGFP* is ectopically activated in suspensor cells, whereas no such expression is observed in the wild type. Together, these complementary approaches support our conclusion that *WOX2* is ectopically expressed in the suspensor of *al* mutants.

6. Figure 5B, D: This figure lacks clarity. The term "occupancy" is undefined, and the number of biological replicates and statistical analyses are not described. The al pentuple mutant appears more affected than the hexuple, which is counterintuitive and questions the reproducibility of the data.
The number of biological replicates and the statistical analyses are now explicitly stated in the Methods. All experiments (CUT&Tag and RNA-Seq) were performed in biological triplicate.

Regarding the use of the term "occupancy", this is the standard terminology used to describe the enrichment of histone modifications across genomic regions in CUT&Tag/ChIP-based datasets. Regarding the comparison between quintuple and sextuple mutants, we would like to clarify that the apparent differences reflect variability inherent to genome-wide datasets rather than a systematic or biologically meaningful discrepancy. For example, in Figure 5D, the total number of H3K4me3 peaks is comparable across genotypes (17,520 in wild type, 19,778 in the quintuple, and 18,659 in the sextuple mutant), indicating no major global shift. Moreover, the overall trends in chromatin changes and gene expression are consistent with the expected role of AL proteins and are in agreement with previously published datasets in seedlings. Finally, to further support the validity of our dataset, we assessed the overlap between H3K4me3 peaks and AL4 genomic binding and observed a strong correlation, indicating that our data capture biologically relevant H3K4me3 patterns.

7. Figure 5E: Sample labels are missing, making the figure uninterpretable.
We agree that the previous presentation of this panel lacked clarity. To improve interpretability, we have redesigned this analysis and now present the correlation scores as box plots, which allow a clearer comparison of similarities and differences across datasets.

8. The proposed antagonistic relationship between H3K4me3 and H3K27me3 is not experimentally tested. Are the same loci affected?
We agree that the previous version of the manuscript overstated the extent of the relationship between H3K4me3 and H3K27me3, and we have revised the text accordingly to avoid implying a general antagonistic mechanism. In our manuscript, we focus on specific loci where both marks can be directly compared. In particular, we present a detailed analysis of the *MAF* gene cluster as case study, where loss of H3K4me3 in *al* mutants is accompanied by a gain of H3K27me3 at the same loci. We therefore restrict our conclusions to these loci and describe this relationship more cautiously as a functional coupling between H3K4me3 and H3K27me3 at a subset of developmental regulators, rather than a global antagonistic interaction.

9. How do the authors link the proposed physical interaction of the FIS-PRC2 with the observed phenotypes of al mutants?
We acknowledge that the structure and wording of the previous version may have suggested a direct functional link between PRC2–AL physical interaction and the observed *al* mutant phenotypes. This was not our intention. In the revised manuscript, we have clarified that we do not claim a direct or functionally relevant interaction between AL proteins and PRC2. Consistent with this, our data show that global H3K27me3 occupancy is not substantially altered in *al* mutants, arguing against a direct role of AL proteins in PRC2 recruitment or activity. Instead, we propose that AL proteins primarily function in the interpretation of H3K4me3 and in maintaining active chromatin states. The observed increase in H3K27me3 at specific loci is therefore interpreted as an indirect consequence of altered chromatin balance, rather than a direct effect on PRC2. In addition, we now include new phenotypic observations showing that the endosperm of *al* sextuple mutants displays defects reminiscent of FIS-PRC2 mutants. We further show that

the expression of the FIS-PRC2 component *MEA* is reduced in *al* sextuple seeds. These findings suggest that some aspects of the developmental phenotypes - particularly those related to endosperm development and embryo patterning - may be partially explained by secondary effects on the expression of PRC2 components. We have revised the manuscript to reflect this more cautious and indirect model.

Referee #2:

The manuscript provides evidence for an epigenetic "crosstalk" between activating and repressive chromatin marks, highlighting the AL protein family's role as H3K4me3 readers that limit H3K27me3 spreading. This work has the potential to address a knowledge gap in plant epigenetics. The authors have integrated IP/MS, RNA-Seq, CUT&Tag, and in situ hybridisation data, which offers a multidimensional perspective. They have also generated higher-order mutants, which enable the functional validation of AL function.

The major weakness is that the study provides limited mechanistic insight, and this should be reflected in the conclusion reached. For example, although AL proteins bind H3K4me3 and correlate with gene activation in developing embryos, the biochemical or structural basis of this interaction is underexplored.
The biochemical and structural basis of H3K4me3 recognition by ALFIN-LIKE proteins has been addressed in previous studies. In particular, Liang et al. (2018, doi.org/10.1007/s10930-018-9796-4) performed a comprehensive biochemical analysis of the PHD domains of all seven AL proteins in *Arabidopsis thaliana*. Using purified proteins and quantitative binding assays, including isothermal titration calorimetry, they demonstrated that AL PHD domains bind H3K4me3 with measurable affinity and defined methylation-state preferences. These findings provide strong biochemical support for the classification of AL proteins as H3K4me3 readers. Given this existing body of work, we consider that further biochemical or structural characterization is beyond the scope of the present study. Instead, our work focuses on the developmental and functional consequences of H3K4me3 interpretation during embryogenesis.

Similarly, it is unclear how ALs distinguish specific target sites or whether they actively recruit the different co-factors in embryos.
ALFIN-LIKE proteins recognize H3K4me3 through their PHD domain, and their chromatin association is therefore expected to largely follow the distribution of this histone modification. To support this, we compared published AL4-GFP ChIP-seq data with our H3K4me3 profiles and observed a strong overlap between AL4 binding sites and H3K4me3-enriched regions in both seedlings and developing seeds. This suggests that AL proteins are broadly associated with H3K4me3-marked chromatin rather than being targeted to specific loci through additional sequence-specific factors. Based on this, we consider it unlikely that AL proteins require dedicated targeting mechanisms beyond H3K4me3 recognition. Instead, their function is more consistent with the interpretation of pre-existing chromatin states. At the same time, we

acknowledge that the precise contribution of additional cofactors or context-dependent interactions remains to be determined and we do not exclude that there is an active recruitment of AL proteins to H3K4me3 marked chromatin.

The "tug-of-war" model between H3K4me3 and H3K27me3 is interesting but lacks temporal chromatin profiling or co-occupancy ChIP experiments to validate competition at the same loci.
We agree that the previous version of the manuscript overstated the “tug-of-war” model without providing direct evidence for co-occupancy or temporal dynamics of H3K4me3 and H3K27me3. We have therefore removed this terminology and revised the manuscript to adopt a more cautious interpretation of the relationship between these marks.
In the revised version, we focus on loci where both modifications can be directly compared. At the MAF gene cluster, loss of H3K4me3 in *al* mutants is accompanied by a gain of H3K27me3 at the same loci, supporting a local relationship between these marks. Importantly, similar patterns have been reported in mutants affecting H3K4me3 deposition. For example, reduced H3K4me3 at the flowering repressor *FLC* in *atx* mutants is associated with increased H3K27me3 (Tamada et al., 2009 doi: 10.1105/tpc.109.070060, Yun et al., 2012 doi.org/10.1093/pcp/pcs021; Pien et al., 2008 doi: 10.1105/tpc.108.058172), and comparable effects have been described at MAF genes and other flowering regulators (Berr et al., 2015, doi.org/10.1111/tpj.12729). These observations suggest that the relationship between reduced H3K4me3 and increased H3K27me3 at flowering regulators is not unique to AL function, but may represent a more general feature of chromatin regulation at these loci. We therefore now describe this as a locus-specific functional coupling between H3K4me3 and H3K27me3, rather than a global antagonistic mechanism.

Similarly, the definition "stochastic epigenetic state" (Line 477) needs more experimental justification as cell-to-cell heterogeneity isn't directly quantified. Some methodological limitations (e.g., lack of cell-type specificity in CUT&Tag from whole seeds) are not discussed.
We agree that the term “stochastic epigenetic state” was too strong given the resolution of our data and the lack of direct single-cell measurements. We have therefore revised the manuscript to use more cautious language and now refer to increased variability in gene expression rather than stochastic epigenetic states. We also acknowledge the limitation of our experimental approach. All chromatin profiling experiments were performed on whole seeds, which include embryo, endosperm, and maternal tissues. As a result, cell-type-specific chromatin changes cannot be resolved in our dataset.

Referee #3:

In this manuscript Scotton et al report a function for the ALFIN H3K4m3-reader proteins in plant development, more precisely embryogenesis and flowering time. They also suggest they function in the dynamic interplay between H3K4m3 and the polycomb repressive complex 2 (PRC2)-mediated H3K27me3. This manuscript complements our understanding of this reader protein family and provides novel aspects. The manuscript is well written and combines developmental

analysis with epigenome profiling in seeds. While the experimental data provided is sound, some of the drawn conclusions are not sufficiently backed by experimental evidence as detailed below. The manuscript has a tendency to report many different (isolated) findings (embryonic phenotypes, flowering phenotype, MAF regulation) and interesting ideas ("tug-of-war"), without tying things into a coherent picture. In addition, the findings of a recent (and cited) publication (Su et al 2025) may suggest alternative explanations in some parts and this needs to be incorporated into the current model.

Specific comments:
1) The idea that ALFINs protect H3K4me3 regions from invading H3K27me3 is very interesting. So far, this is shown on a "count" basis (genes gaining or losing modifications). It would be helpful to follow this up showing average gene plots of the different groups. It also needs to be clarified whether the modification "spreads" from neighboring regions that are enriched in H3K27me3 (as the heading in line 444 suggests) or whether it is newly laid down in the genes that loose H3K4me3. The MAF screenshots in Fig. 6 are only of limited help. To me the gain in H3K27me3 is limited and very localized, and the loss in H3K4me3 appears even smaller.
We agree that the previous version of the manuscript overinterpreted the relationship between H3K4me3 and H3K27me3 and did not sufficiently resolve whether H3K27me3 spreading or de novo deposition occurs. Addressing this question would require high-resolution, embryo-specific chromatin profiling across developmental stages and genetic backgrounds, which remains technically extremely challenging in *Arabidopsis* due to the small size of early embryos. In the revised manuscript, we have therefore removed the “tug-of-war” model and now present a more cautious interpretation of the data. Our genome-wide analysis identifies only a limited number of loci showing concurrent loss of H3K4me3 and gain of H3K27me3. At present, it is unclear whether this reflects biological specificity or technical limitations inherent to chromatin profiling in complex tissues such as seeds. Given the relatively small number of affected loci, we consider that global metagene analyses would not provide meaningful additional insight. Instead, we focused on the *MAF* gene cluster as a representative case, where all three criteria are met: loss of H3K4me3, gain of H3K27me3, and reduced expression in the RNA-seq dataset. This allows a more robust locus-specific analysis compared to global trends. We have revised the figure and text to better present this case study. Interestingly, similar relationships between reduced H3K4me3 and increased H3K27me3 at flowering regulators have been reported in mutants affecting H3K4me3 deposition (e.g., *atx* mutants; Berr et al., 2015, doi.org/10.1111/tpj.12729).

2) It is a vey exciting idea that H3K4me3/ALFIN mediates robust gene expression levels and that stochastic changes appear in the mutant. The in situ hybridization pictures are suggestive but not convincing. It looks like the expression in the heart stage embryo (6d) may simply be stronger in the inner cell layers of the cotyledons. Similarly, this pattern can be seen in (6c late heart stage embryo). The authors should provide more (convincing) pictures. Also, a sense probe as negative control is missing. It would be nice to link this phenotype also to genes

relevant for embryo development, such as the mentioned WOX2 and others. This would strengthen the conclusion of enhanced stochastic variation in gene expression in alfin mutants.
In the revised manuscript, we have moderated our interpretation and no longer refer to stochastic gene expression. Instead, we describe the phenotype more cautiously as increased variability in expression patterns in *al* mutants. In addition, we provide additional *in situ* hybridization images with improved signal quality, together with a sense probe control. These consistently show that, while expression in wild-type embryos remains uniform, *al* mutants display patchy and heterogeneous expression patterns across embryos.
We acknowledge that *in situ* hybridization is not a quantitative technique and does not allow rigorous assessment of cell-to-cell variability. Furthermore, while the patchy expression pattern is reproducibly observed in *al* mutants, it is not fully penetrant across all embryos. We therefore limit our conclusions to a qualitative increase in variability rather than a strict demonstration of stochastic gene expression. We agree that extending this analysis to additional markers could further support this observation. However, given the limitations of *in situ* hybridization in quantifying expression variability, we believe that additional experiments of the same nature would not substantially strengthen the mechanistic interpretation. A more rigorous assessment of stochastic gene expression would require complementary approaches beyond the scope of the present study.

3) While FLC expression is reset during early embryogenesis, there is no evidence that embryonic FLC expression is crucial for flowering time control. The discussion leaves a wrong impression and needs correction. It is thus a long stretch from a possibly reduced (?) MAF expression in early embroys to a late flowering phenotype. The authors need to show the altered expression level in seedlings. Su et reported epigenomic analysis of relevant modifications in seedlings of alfin mutants. Did the authors check whether they hypothesis for MAF gene expression is supported by those data sets?
We agree that the previous version of the manuscript overstated the link between embryonic gene expression and flowering time. In the revised manuscript, we have moderated our conclusions and now avoid implying a direct causal relationship between embryonic *MAF* expression and adult flowering time. Following the reviewer's suggestion, we examined publicly available datasets from Su et al. (2025) and found that multiple AL proteins (AL1, AL2, AL3, AL4, and AL6) directly associate with *MAF* loci. In addition, in *al* higher-order mutants, *MAF* genes show coordinated chromatin changes, including loss of H3K4me3 (*MAF1*, *MAF2*) and gain of H3K27me3 (*MAF1*, *MAF2*, *MAF4*). These observations indicate that AL proteins directly regulate the chromatin state of flowering-related genes, both in embryos and seedlings.

It is an interesting question whether developmental time is counted in days or in leaves. To conclude that reduced embryonic MAF expression and early flowering fits together is cherry picking, as with the same right you could say the plants are late flowering. In this respect, an increased plastochron is interesting, given that the rosettes over all do not look smaller - judging from Fig EV1b. Additional phenotypic information would be useful. The legend of this Fig should also have a size bar and specify the age and growing conditions of the plants.

The suggested misexpression of MAFs and the flowering phenotype however remain at the stage of correlation, as any number of flowering regulators might similarly or even more clearly misexpressed. - I understand that it would require to stack a maf quadruple mutant onto the alfin sextuple mutant to test this, so I am not suggesting it.
Flowering time in our study was already quantified both as days to bolting and as rosette leaf number at bolting (Fig. 7). We agree that these two measures capture distinct aspects of development, and we have revised the manuscript to make this distinction more explicit. Importantly, *al* mutants display a dissociation between chronological and developmental flowering time: while bolting is delayed in terms of days, it occurs at a reduced rosette leaf number meaning this mutant does not exhibit a simple “early” or “late” flowering phenotype. In this context, the reduced leaf number at bolting indicates that flowering competence is reached at an earlier developmental stage, whereas the delayed bolting in days reflects overall slower growth. We have clarified this interpretation in the revised Results section to avoid ambiguity. We therefore respectfully disagree that the interpretation represents cherry picking. Instead, the divergence between these two commonly used metrics is itself informative and reveals an alteration in developmental timing rather than a unidirectional shift in flowering time.
We agree with the reviewer that the current data do not establish a causal link between reduced embryonic *MAF* expression and the flowering phenotype. We have accordingly tempered our conclusions throughout the manuscript and now explicitly state that this relationship is correlative. Regarding the plastochron and rosette appearance, we note that overall rosette size appears comparable at bolting stage despite the altered leaf initiation rate, and have provided more informative images in Figure 7. However, we also noticed that leaves keep growing in dimension after flowering of the plant, and utlimately they became very big, sometime as twice the size as wild type plants.

4) Fig 5a-c: H3K4me3 is typically enriched at the TSS. The squeezing of gene bodies in Fig 5a and c in not suited to judge whether there is mislocalization of the modification in alfin mutants.
We agree that metagene representations have limitations in resolving fine-scale changes in the distribution of histone modifications along gene bodies. However, the purpose of this analysis was not to assess subtle redistribution within gene bodies, but rather to evaluate global patterns of H3K4me3 enrichment. As visible from the average profiles (blue lines), H3K4me3 remains strongly enriched at the transcription start site (TSS) in both wild-type and *al* mutant backgrounds, with no major shift or spreading into gene bodies. We have clarified this point in the revised manuscript and now explicitly state that we do not observe evidence for large-scale mislocalization of H3K4me3 in *al* mutants. Instead, our analysis focuses on locus-specific changes in H3K4me3 levels.

5) The manuscript would be strengthened considerable if it was known which of the genes that alter chromatin modifications are direct targets of ALFINs. While it may be difficult to globally determine ALFIN-bound genes, CHIP-seq for the 1-2 most relevant ALFINs should be done to be able to clarify whether the affected genes are direct target genes.

As discussed above, performing ChIP-seq specifically in early embryos presents substantial technical challenges due to the limited amount of material and the broad expression of AL proteins across seed tissues. This makes it difficult to obtain sufficient, cell-type-specific chromatin profiles with appropriate replication and controls.
To address this point, we took advantage of publicly available AL4-GFP ChIP-seq datasets generated in seedlings (Su et al., 2025). By comparing these data with our H3K4me3 profiles, we observed a strong overlap between AL4 binding sites and H3K4me3-enriched regions genome-wide. Furthermore, a substantial fraction of loci showing altered chromatin states in our dataset are associated with AL4-bound regions in these independent datasets.
While we acknowledge that these data are derived from a different developmental context, they support the conclusion that AL proteins are broadly associated with H3K4me3-marked chromatin and suggest that many of the affected loci are likely direct targets.
We have now incorporated this analysis and explicitly discussed this limitation in the revised manuscript.

6) tug-of-war is a catchy phrase. I am not convinced it is appropriate here, as the effect of loss of H4K27me3 on K4me3 is not studied.
We have removed the “tug-of-war” terminology from the manuscript and revised the text to provide a more cautious and accurate description of the relationship between H3K4me3 and H3K27me3. Our conclusions are now restricted to the observed changes in H3K4me3 and H3K27me3 in *al* mutants, without implying reciprocal or competitive interactions.

7) A role in robustness (reducing stochastic variation) in gene expression has been reported for the Paf1c complex (Fal et al, 2019; doi:10.1242/dev.173377) . Maybe ALFINs are involved with Paf1c?
We thank the reviewer for this suggestion. Interestingly, mutants in components of the Paf1C complex, such as *vip3*, display developmental phenotypes that partially resemble those observed in higher-order *al* mutants, including alterations in fruit development and aspects of tissue identity. In addition, both systems are associated with deregulation of MADS-box and homeobox genes, suggesting a potential overlap at the level of transcriptional regulation.
These parallels raise the possibility that AL proteins and Paf1C may act in related or converging pathways. However, the potential mechanistic link between these factors has not been explored and falls beyond the scope of the present study.

8) Su et a 2025 report a connection between ALFIN and PRC1/H2A ub, as well as a direct interaction with the K27me3 demethylase REF6. The latter is also in the list of ALFIN interactors presented here. Could this be a major mechanism of antagonizing H3K27me3? Could the effects on PRC2/H3K27me3 be indirectly mediated or supported by its effect on PRC1? None of this is discussed in this manuscript although it is highly relevant for the interpretation of the data.
In the revised manuscript, we have expanded the Discussion to include these aspects. In particular, we now discuss the potential involvement of PRC1/H2Aub and the H3K27me3 demethylase REF6, both of which have been linked to ALFIN proteins in recent work (Su et al.,

2025). We note that REF6 was also identified in our IP/MS datasets, further supporting a possible functional connection. While our data do not allow us to directly test these mechanisms, these observations raise the possibility that the effects on H3K27me3 observed in *al* mutants may be mediated, at least in part, through indirect pathways involving PRC1 or H3K27me3 demethylation rather than direct modulation of PRC2 activity. We have now incorporated this perspective into the revised Discussion to provide a more comprehensive framework for interpreting our findings.

July 10, 2026

RE: Life Science Alliance Manuscript #LSA-2026-03835-T

Prof. Sara Simonini
University of Zurich
Institute of Plant and Microbial Biology
Zollikerstrasse 107
Zurich CH8008
Switzerland

Dear Dr. Simonini,

Thank you for submitting your revised manuscript entitled "ALFIN-LIKE H3K4me3 Reader Proteins Regulate Embryogenesis in Arabidopsis".

In accordance with our offer to publish this work, please provide a revised manuscript with the following changes:
-Adjust the description of IP-MS results by specifying the Trithorax-related regulators, SWI/SNF-associated components, and PRC1-related components identified, and remove or clarify reference to proteins falling below the significance threshold. Soften the claims related to these findings as appropriate.
-Improve accuracy in describing specific results (Reviewer 3 points 4-7)
-Acknowledge previous work (Reviewer 3 point 9)

We would be happy to publish your paper in Life Science Alliance pending resolution of the above points and final revisions necessary to meet our formatting guidelines.

MANUSCRIPT ORGANIZATION AND FORMATTING:

To avoid unnecessary delays in the acceptance and publication of your paper, please read the following information carefully. Full guidelines are available on our Instructions for Authors page, https://www.life-science-alliance.org/authors

Submission of a paper that does not conform to Life Science Alliance guidelines will delay the acceptance of your manuscript.

-Thank you for providing a data availability statement. Please expand to include protein mass spectrometry datasets and refer to LSA's guidelines on data availability here https://www.life-science-alliance.org/editorial-policies#data-sharing
-We suggest you to include if any stocks or constructs generated in the study have been deposited in any public repositories such as Arabidopsis Biological Resource Center (ABRC).
-Kindly expand on imaging details to include objectives for all imaging experiments and excitation and emission filters for fluorescence imaging.
-Please upload your main manuscript text as an editable doc file.
-Please upload all figure files as individual ones, including the supplementary figure files; all figure legends should only appear in the main manuscript file. Please remove all figures from main manuscript file.
-Please add a Running Title in our system.
-please add Keywords for your manuscript in our system.
-Please add a Category for your manuscript in our system.
-Please add the X and Bluesky handles of your host institute/organization, as well as your own, and/or one of the authors, in our system.
-Please add a Summary Blurb/Alternate Abstract in our system
-Please make sure that all Supplementary Tables are in xlsx file format and labeled as Supplementary Table S1,S2 etc. Callouts in the manuscript need to be adjusted as well.
-Conflict of interest statement in manuscript should be labeled as such.
-Please remove section titled, "SUPPLEMENTAL INFORMATION Supplemental Information can be found online at ..."
-Please use the [10 author names et al.] format in your references (i.e., limit the author names to the first 10).
-Please add your main, supplementary figure, and table legends to the main manuscript text after the references section; Please name the sections as: 'Figure legends", "Supplementary figure legends" and "Supplementary Tables legends".
-LSA allows supplementary figures, but no EV Figures; please update your callouts for the Supplementary Figures in the manuscript Fig EV1A=Fig S1A; Supplementary figures should be labeled as supplementary Figure S1.
-Figure EV2 (S2): please make sure that panels in the figure correspond to the panels in the caption (I-K not listed in the captions); please update the callouts as well.
-Please be sure that the authorship listing and order is correct

We encourage our authors to provide original source data, particularly uncropped/-processed electrophoretic blots and spreadsheets for the main figures of the manuscript. If you would like to add source data, we would welcome one PDF/Excel-file per figure for this information. These files will be linked as supplementary "Source Data" files.

It is Life Science Alliance policy that if requested, original data images must be made available to the editors. Failure to provide original images upon request will result in unavoidable delays in publication. Please ensure that you have access to all original data images prior to final submission.

If you are planning a press release on your work, please inform us immediately to allow informing our production team and scheduling a release date.

We welcome submissions of potential cover images for the issue of LSA in which your work would appear. If you have high quality images associated with this work, please feel free to email these, with a caption, to the journal office.

LSA encourages authors to provide a 30-60 second video where the study is briefly explained. These videos will be appear embedded with the manuscript online at Life Science Alliance, and on social media to promote the published paper and authors (for examples, see https://docs.google.com/document/d/1-UWCfbE4pGcDdcgzcmiuJl2XMBJnxKYeqRvLLrLSo8s/edit?usp=sharing). Corresponding or first-authors are welcome to submit the video. Please submit only one video per manuscript. The video can be emailed to contact@life-science-alliance.org

FINAL FILES:

To upload the final version of your manuscript, please log in to your account: https://lsa.msubmit.net/cgi-bin/main.plex
You will be guided to complete the submission of your revised manuscript and to fill in all necessary information. Please get in touch in case you do not know or remember your login name.

The following items are required for acceptance.

-- An editable version of the final text (.DOC or .DOCX) is needed for copyediting (no PDFs).

-- High-resolution figure, supplementary figure and video files uploaded as individual files: See our detailed guidelines for preparing your production-ready images, https://www.life-science-alliance.org/authors

-- Summary blurb (enter in submission system): A short text summarizing in a single sentence the study (max. 200 characters including spaces). This text is used in conjunction with the titles of papers, hence should be informative and complementary to the title. It should describe the context and significance of the findings for a general readership; it should be written in the present tense and refer to the work in the third person. Author names should not be mentioned.

The license to publish form must be signed before your manuscript can be sent to production. A link to the license to publish form will be available to the corresponding author only. Please take a moment to check your funder requirements.

Reviews, decision letters, and point-by-point responses associated with peer-review at Life Science Alliance will be published, alongside the manuscript. If you do want to opt out of having the reviewer reports and your point-by-point responses displayed, please let us know immediately.

Thank you for your attention to these final processing requirements. Please revise and format the manuscript and upload materials as soon as you are able.

Thank you for this interesting contribution to the literature. We look forward to publishing your paper in Life Science Alliance.

Sincerely,

Sarita Hebbar, PhD
Scientific Editor
Life Science Alliance
http://www.lsajournal.org

Response to reviwers and Editor comments

Referee #1:

Unfortunately, I do not find that the manuscript has substantially improved. In addition to my continuing concerns regarding the interaction data, as detailed below in comments 1 and 2, many of the conclusions remain overstated. Also the novelty of the study is limited in light of two recent publications that have addressed both the mechanism of AL function and the functional requirement of AL proteins during vegetative and reproductive development, including flowering-time control (Su et al., 2025; Xu et al., 2025). Although these studies are cited, the manuscript does not clearly explain what new insight the present work provides relative to this existing knowledge.

We thank the reviewer for their careful assessment of the revised manuscript and for their comments, which have helped us further clarify the scope and conclusions of the study. We agree that two important studies on ALFIN-LIKE proteins have recently been published (Su et al., 2025; Xu et al., 2025), and, as the reviewer notes, these studies are extensively cited and discussed in the revised manuscript. We also agree that some of our findings provide additional information on ALFIN-LIKE proteins that, considered individually, may not constitute major conceptual advances. However, we respectfully disagree that the reproductive defects characterized in our study have already been reported in these previous studies. Both Su et al. (2025) and Xu et al. (2025) report the difficulty, or inability, to recover the complete *al* septuple mutant, consistent with an essential function of AL proteins during reproduction. These manuscripts additionally show reduced silique length and interpret this phenotype as evidence for a role of AL proteins in reproduction. These observations establish that AL proteins are required for successful reproduction, but they do not identify which reproductive process is affected. Plant reproduction encompasses several distinct developmental processes, including male and female gametophyte development, fertilization, embryogenesis, and endosperm development, and reduced silique length alone cannot distinguish among defects occurring at these different stages. Our study extends these observations by defining the developmental basis of the reproductive phenotype. We characterize the defects during early seed development and show that loss of AL function affects zygote elongation and the first asymmetric division, disrupts apical–basal patterning and cell identity, and leads to abnormal embryonic development. Thus, rather than simply providing further evidence that AL proteins have a role in reproduction, our work identifies the specific developmental processes affected and links AL function to the establishment of embryonic patterning during the earliest stages of seed development.

1. Related to Comment 1: Rather than generating higher-quality data to address the concern, the authors have reanalyzed the existing dataset. However, even after this reanalysis, the proposed interaction between AL4 and PRC2 subunits is not convincingly supported. The claimed interactions do not appear to pass the significance threshold. Moreover, the interaction data for AL4 could not be properly assessed because the relevant proteins were not annotated in the Supplementary table. Although the authors state that the AL-PRC2 interaction is not the main focus of the study, they nevertheless present and discuss these data in the manuscript. Given the quality and limitations of the dataset, I do not think this is justified.

We would like to clarify that the claim of an interaction between AL4 and PRC2 referred to by the reviewer was present in Version 1 of the manuscript. In response to the reviewer's original concern, we removed this interpretation from Version 2 and revised the corresponding text specifically to avoid implying that our data demonstrate a physical interaction between AL4 and PRC2 components. We agree that the available dataset does not provide sufficient evidence to establish such an interaction, and Version 2 does not make this claim. The revised text makes this distinction explicit. The data are not presented as evidence for an AL4–PRC2 interaction, and we do not draw this conclusion from them. We hope that this clarification resolves the apparent contradiction between the reviewer's comment and the conclusions presented in the revised manuscript.

2. Related to Comment 2: My concern remains the same as for comment 1. The data provided do not justify discussion of this interaction. In addition, the statement in the response letter "Instead, we interpret the IP/MS results as reflecting the presence of these factors in overlapping chromatin-associated environments, particularly during early seed development" is unclear. It is not evident what specific conclusion the authors intend to draw from this interpretation, nor how it is supported by the data.
We thank the reviewer for pointing this out. As clarified in our response to Comment 1 above, Version 2 of the manuscript no longer interprets the IP-MS data as evidence for a physical interaction between AL4 and PRC2 components.
We also agree that the statement in our previous response letter that the IP-MS results might reflect "the presence of these factors in overlapping chromatin-associated environments" was unclear and went beyond what can be concluded from the data. Our intention was simply to explain why PRC2-associated proteins might have been detected in the AL4 IP-MS experiment without implying a direct interaction. However, we agree that the IP-MS data alone do not allow us to distinguish among possible explanations for their detection. We therefore withdraw this interpretation and do not draw any conclusion regarding an AL4–PRC2 interaction or association from these data.

3. Related to Comment 3: The additional reporter lines are a useful addition. However, a negative control is required to distinguish the weak signals observed in the egg and central cell from background signal.
We respectfully disagree that the GFP signals observed in the egg and central cell are weak or require an additional negative control for their interpretation. As evident from the images presented in the manuscript, the GFP fluorescence is strong, clearly distinguishable from background fluorescence, and spatially restricted within the ovule. The surrounding non-expressing tissues within the same sample provide an internal reference for background fluorescence. We therefore consider the reporter expression in the egg and central cell to be unambiguous and do not believe that an additional negative control is required to support this conclusion.

4. Page 3: The conclusion that "H3K4me3 is not uniformly distributed across active genes, but is preferentially associated with developmental regulators during embryogenesis" is not supported by the data presented. To substantiate this conclusion, the authors would need to test whether the occurrence of H3K4me3 is independent of, or not simply correlated with, transcriptional activity.

We respectfully disagree that this conclusion requires demonstrating that H3K4me3 is independent of transcriptional activity. Our analysis specifically controls for transcriptional activity by restricting the comparison to genes identified as expressed by RNA-seq. We then asked whether, within this population of active genes, H3K4me3-marked genes represent a random subset with respect to gene function, or whether particular functional categories are preferentially represented. Developmental regulators were significantly enriched among the H3K4me3-marked active genes relative to their expected representation within the active-gene population. Thus, the conclusion does not imply that H3K4me3 occurs independently of transcription; rather, it shows that among transcriptionally active genes, H3K4me3 is preferentially associated with genes involved in developmental regulation. To ensure that the scope of this conclusion accurately reflects the biological material analysed, we have also revised the text from "during embryogenesis" to "during seed development." The sentence now reads: "*These findings indicate that H3K4me3 is not uniformly distributed across active genes, but is preferentially associated with developmental regulators during seed development, suggesting that its genomic allocation is dynamically reconfigured to support early patterning programs."*

5. The transcriptome analysis was performed on seeds at 1-4 DAP, and the authors state that this stage is "before the most pronounced morphological defects arise in al mutants." However, based on Figure 3h, embryo arrest occurs at the globular stage, which corresponds to the 1-4 DAP window.

We agree with the reviewer that the 1–4 DAP window overlaps with the period during which morphological defects become evident in *al* mutants. We have therefore revised the text to accurately reflect this, as follows: "*a developmental window during which morphological defects become evident in al mutants."*

6. Page 11: The authors used siliques for the IP-MS experiment, but then conclude that "IP/MS analysis revealed a robust AL4-associated chromatin interactome in developing seeds." Because the input material was whole siliques, conclusions specifically about seeds cannot be drawn.

We agree with the reviewer and have revised the text accordingly, replacing "developing seeds" with "developing siliques" to accurately reflect the material used for the IP-MS analysis.

7. Page 13: Any inferences about the co-occurrence of H3K4me3 and H3K27me3 should be removed or substantially toned down. The authors used silique tissue, which contains multiple distinct cell types. Therefore, the data do not allow reliable conclusions about co-occurrence of these marks in a biologically meaningful way.

We would like to clarify that these analyses were performed on whole seeds, not siliques. Nevertheless, we agree that whole seeds comprise multiple cell types and have revised the text to explicitly acknowledge that the observed overlap cannot distinguish co-occurrence within individual cells from the presence of the two marks in different cell populations. We have therefore limited our conclusion to the reduced segregation of H3K4me3- and H3K27me3-associated genomic regions in seeds compared with seedlings. The text now reads as follow "*Because these analyses were performed on whole seeds, which comprise multiple cell types with distinct chromatin states, this increased overlap cannot distinguish between co-occurrence of the two marks within individual cells and their presence in different cell*

populations. Nevertheless, it indicates that the genomic distributions of H3K4me3- and H3K27me3-associated regions are considerably less segregated in seeds than in seedlings, consistent with the dynamic chromatin landscapes of early seed development."

8. Page 15: The claimed more heterogeneous and patchy expression of MAF genes at later stages is not apparent from the data provided.
We appreciate the reviewer's comment. In situ hybridization provides a qualitative assessment of spatial expression rather than a quantitative measurement. In our analysis, *al1/3/4/5/6/7* embryos consistently showed staining restricted to different regions of the embryo, which we consider best described as a "patchy" expression pattern. Additional examples are provided in the Supplementary Figure. Importantly, we did not observe a comparable pattern in wild-type embryos, in which the staining was consistently more uniform. We have retained this terminology as we believe it most accurately describes the observed spatial distribution of the signal.

9. Finally, the late-flowering phenotype of al mutants has already been reported previously by Su et al. (2025). This should be clearly acknowledged, and the manuscript should avoid presenting this observation as a novel finding.
We agree that the association between ALFIN-LIKE proteins and flowering time has previously been reported by Su et al. (2025). This study was already discussed and cited in the manuscript, and our text did not intend to present the late-flowering phenotype as a novel finding. To make this even more explicit, we have now added the citation directly to the sentence introducing our flowering-time analysis: "*Given the reported association between ALFIN-LIKE proteins and flowering time (Su et al., 2025), we next quantitatively assessed flowering behaviour in the al1/3/4/5/6/7 sextuple mutant using both days to bolting and rosette leaf number at the time of floral transition. Consistent with the previous report (Su et al., 2025), sextuple mutants bolted slightly later than wild type. However, mutant plants also produced substantially fewer but enlarged rosette leaves prior to flowering (Fig. 7D–G).*"

Referee #2:

The manuscript has improved significantly. The authors have addressed all my concerns and most of the issues raised by other reviewers. In my opinion, the manuscript is suitable for publication in this journal.
We thank the reviewer for their positive assessment of the revised manuscript and for their constructive comments throughout the review process.

Referee #3:

In this new version of the manuscript the authors removed the exaggerated claims they made in the original version (tug or war, stochastic variation on gene expression, interaction with PRC2) that were critiqued by myself and the other reviewers. The authors chose to remove them rather than providing additional experimentation to support their claims. The revised manuscript is an interesting, yet somewhat descriptive characterization of the function of AL

proteins during embryogenesis; crucially, it does not add to our understanding of what AL proteins are doing mechanistically and molecularly. The experiments are now technically sound and the remaining conclusions justified. However, the manuscript still lacks focus. In summary, due to the lack of mechanistic insights, the manuscript would be better suited for a more specialized journals.

We thank the reviewer for their assessment of the revised manuscript and for acknowledging that the experiments are technically sound and that the conclusions of the revised manuscript are justified. We appreciate the reviewer's comments throughout the revision process, which helped us refine the scope and interpretation of the study.

Comments from the Editor:

-Adjust the description of IP-MS results by specifying the Trithorax-related regulators, SWI/SNF-associated components, and PRC1-related components identified, and remove or clarify reference to proteins falling below the significance threshold. Soften the claims related to these findings as appropriate.

We have revised the description of the IP-MS results accordingly. We now explicitly identify the Trithorax-related regulator ATX2, the SWI/SNF-associated components BRM, ARP7, SWI3A, SWI3B and SWI3C, and the PRC1-associated/repressive chromatin regulators RING1A, VAL1, HDA19 and MSI1 recovered in AL4 immunoprecipitates. We have also clarified the statistical support for the reciprocal IP-MS analyses, distinguishing proteins that were significantly enriched from those that were detected but did not reach the significance threshold. In particular, AL4 was significantly enriched in MEA immunoprecipitations, whereas AL7 was detected but did not reach significance; AL7 was significantly enriched in SWN immunoprecipitations. Finally, we have softened the interpretation of these results and do not present the IP-MS data as evidence for a direct interaction between AL proteins and PRC2 components.

Now the text reads: *"IP/MS analysis identified a robust AL4-associated chromatin protein network in developing siliques (Fig. 5A; Supplementary Table S3). As expected based on previous studies in seedlings (Su et al., 2025), AL4 co-purified with numerous factors involved in transcriptional activation and chromatin remodeling, including the Trithorax-related histone methyltransferase ATX2 and multiple SWI/SNF chromatin-remodeling components, such as BRAHMA (BRM), ARP7 and the SWI3A, SWI3B and SWI3C subunits, consistent with a conserved role of AL proteins within activating chromatin environments. AL4 immunoprecipitates also contained proteins previously associated with transcriptional repression, including the PRC1 component RING1A (Molitor et al., 2014), the B3-domain transcriptional repressor VAL1, the histone deacetylase HDA19 and MSI1. Together, these findings indicate that AL4 is recovered in chromatin-associated protein complexes containing both activating and repressive chromatin regulators during early silique development.*

Reciprocal IP-MS analyses of GFP-tagged PRC2 methyltransferases likewise recovered AL proteins among MEA-associated protein complexes. In developing siliques, the seed-specific methyltransferase MEA significantly co-purified with AL4, whereas AL7 was detected but did not reach the significance threshold (Fig. 5B; Supplementary Table S3). By contrast, AL7 was significantly enriched in SWN immunoprecipitations (Fig. 5D–F; Supplementary Table S3), indicating that recovery of AL proteins is not restricted to a single PRC2-associated dataset."

-Improve accuracy in describing specific results (Reviewer 3 points 4-7).

We have revised the manuscript to improve the accuracy of the results described in Reviewer 3 points 4–7. Specifically, we clarified the interpretation and developmental scope of the H3K4me3 analysis, accurately described the 1–4 DAP RNA-seq window as the period during which morphological defects become evident, corrected the IP-MS material from developing seeds to developing siliques, and clarified the interpretation of H3K4me3/H3K27me3 overlap in whole seeds, explicitly acknowledging that these data cannot distinguish co-occurrence within individual cells from occurrence in different cell populations.

-Acknowledge previous work (Reviewer 3 point 9).

The reviewer refers to the work of Su et al. (2025). This work was already extensively acknowledged and discussed throughout the manuscript and was cited 14 times in the previous version. Nevertheless, to eliminate any possible ambiguity regarding the novelty of the flowering-time phenotype, we have now added two additional citations directly within the section describing our flowering-time analysis and explicitly state that the association between ALFIN-LIKE proteins and flowering time has been previously reported.

The text now reads (added citations in **bold**): "Analysis *of published datasets (Su et al., 2025) shows that MAF loci are associated with AL proteins and display reduced H3K4me3 together with increased H3K27me3 in post-embryonic tissues (Su et al., 2025). The observation of comparable chromatin changes at these loci in both embryos and seedlings indicates that the effects of AL loss are not restricted to early developmental stages, but are also present at later stages of development. Given the reported association between ALFIN-LIKE proteins and flowering time* ***(Su et al., 2025),*** *we next quantitatively assessed flowering behaviour in the al1/3/4/5/6/7 sextuple mutant using both days to bolting and rosette leaf number at the time of floral transition. Consistent with the previous report* ***(Su et al., 2025****), sextuple mutants bolted slightly later than wild type. However, mutant plants also produced substantially fewer but enlarged rosette leaves prior to flowering (Fig. 7D–G)."*

September 29, 2026

RE: Life Science Alliance Manuscript #LSA-2026-03835-TR

Prof. Sara Simonini
University of Zurich
Institute of Plant and Microbial Biology
Zollikerstrasse 107
Zurich CH8008
Switzerland

Dear Dr. Simonini,

Thank you for submitting your Research Article entitled "ALFIN-LIKE H3K4me3 Reader Proteins Regulate Embryogenesis in Arabidopsis". It is an enormous pleasure to let you know that your manuscript is now accepted for publication in Life Science Alliance. Congratulations on this interesting work.

The final published version of your manuscript will be deposited by us to PubMed Central upon publication.

Your manuscript will now progress through copyediting and proofing. Your article will publish open access upon publication under a CC-BY license.

Reviews, decision letters, and point-by-point responses associated with peer-review at Life Science Alliance will be published, alongside the manuscript. If you do want to opt out of having the reviewer reports and your point-by-point responses displayed, please let us know immediately.

IMPORTANT: If you will be unreachable at any time, please provide us with the email address of an alternate author. Failure to respond to routine queries may lead to unavoidable delays in publication.

Scheduling details will be available from our production department. You will receive proofs shortly before the publication date. Only essential corrections can be made at the proof stage so if there are any minor final changes you wish to make to the manuscript, please let the journal office know now.

DISTRIBUTION OF MATERIALS:
Authors are required to distribute freely any materials used in experiments published in Life Science Alliance. Authors are encouraged to deposit materials used in their studies to the appropriate repositories for distribution to researchers.

You can contact the journal office with any questions, contact@life-science-alliance.org

Again, congratulations on a very nice paper. I hope you found the review process to be constructive and are pleased with how the manuscript was handled editorially. We look forward to future exciting submissions from your lab.

Sincerely,

Tim Fessenden
Scientific Editor
Life Science Alliance
